# Homologous Boosting with Adenoviral Serotype 5 HIV Vaccine (rAd5) Vector Can Boost Antibody Responses despite Preexisting Vector-Specific Immunity in a Randomized Phase I Clinical Trial

**DOI:** 10.1371/journal.pone.0106240

**Published:** 2014-09-29

**Authors:** Uzma N. Sarwar, Laura Novik, Mary E. Enama, Sarah A. Plummer, Richard A. Koup, Martha C. Nason, Robert T. Bailer, Adrian B. McDermott, Mario Roederer, John R. Mascola, Julie E. Ledgerwood, Barney S. Graham, the VRC 015 study team

**Affiliations:** 1 Vaccine Research Center, National Institute of Allergy and Infectious Diseases, National Institutes of Health, Bethesda, MD, United States of America; 2 Biostatistics Research Branch, Division of Clinical Research, NIAID, NIH, Bethesda, MD, United States of America; Sanaria, Inc., United States of America

## Abstract

**Background:**

Needle-free delivery improves the immunogenicity of DNA vaccines but is also associated with more local reactogenicity. Here we report the first comparison of Biojector and needle administration of a candidate rAd5 HIV vaccine.

**Methods:**

Thirty-one adults, 18–55 years, 20 naive and 11 prior rAd5 vaccine recipients were randomized to receive single rAd5 vaccine via needle or Biojector IM injection at 10^10^ PU in a Phase I open label clinical trial. Solicited reactogenicity was collected for 5 days; clinical safety and immunogenicity follow-up was continued for 24 weeks.

**Results:**

Overall, injections by either method were well tolerated. There were no serious adverse events. Frequency of any local reactogenicity was 16/16 (100%) for Biojector compared to 11/15 (73%) for needle injections. There was no difference in HIV Env-specific antibody response between Biojector and needle delivery. Env-specific antibody responses were more than 10-fold higher in subjects receiving a booster dose of rAd5 vaccine than after a single dose delivered by either method regardless of interval between prime and boost.

**Conclusions:**

Biojector delivery did not improve antibody responses to the rAd5 vaccine compared to needle administration. Homologous boosting with rAd5 gene-based vectors can boost insert-specific antibody responses despite pre-existing vector-specific immunity.

**Trial Registration:**

Clinicaltrials.gov NCT00709605 NCT00709605

## Introduction

The use of needle-free injection devices for administration of vaccines is a useful and safe alternative method of vaccine delivery. Although associated with slightly more local reactogenicity this technique offers the advantage of eliminating the risk of needle stick accidents in the healthcare setting. The Biojector utilizes single use cartridges for intramuscular administration hence removing the possibility of transferring blood borne pathogens between individuals. In addition to its safety advantages, Biojector administration of vaccines has been associated with improved immune responses in both preclinical as well as clinical studies [1]–[3]. More than a decade ago others had shown that immunization of rabbits with a malaria DNA vaccine via Biojector resulted in improved antibody titers compared with needle injections [2]. In one of the earlier clinical studies that compared routes of administration, higher rates of seroconversion as well as higher antibody titers were seen in response to a hepatitis A vaccine in the Biojector group as compared to needle and syringe (N/S) groups [4].

The Vaccine Research Center (VRC) has extensive experience with use of Biojector for administration of plasmid DNA vaccines. Biojector administration has been utilized for evaluating vaccines for SARS, West Nile virus, influenza and HIV previously [5]–[9]. The results of VRC 008, a Phase I study comparing HIV DNA vaccine delivery by Biojector versus N/S followed by rAd5 (recombinant adenoviral serotype 5 vector vaccine) boost showed that delivery of HIV DNA vaccine by Biojector significantly improved both humoral and cellular immune responses post boost and that the magnitude of immune responses is affected by the method of delivery [10].

The study described in this report was conducted concurrently with HVTN 505, a large Phase 2b efficacy trial evaluating VRC's HIV DNA prime and rAd5 vaccine boost regimen. One of the purposes of the current study was to answer important scientific questions about the administration of the rAd5 vaccine to prepare for the HVTN 505 study outcome. The outcome of HVTN 505 study showing no efficacy at protection from HIV infection precludes any further testing of this vaccine regimen [11]. However, the data from our trial are informative for other vaccine programs for *Plasmodium falciparum*, *Leishmania*, *Trypanosoma cruzi*, dengue virus, influenza, Ebola and others utilizing recombinant adenoviral vectors [12]–[16].

Data from a previous clinical trial that evaluated a homologous rAd5 HIV vaccine regimen showed that boosting with the same viral vector increased Env-specific antibody responses but did not increase T cell responses [17]. In our study, we included subjects who had been previously vaccinated with the rAd5 HIV vaccine to assess whether pre-existing vector-induced Ad5-specific antibody had any effect on antibody responses to the recombinant Env antigen. The molecular targets of neutralization differ if Ad5 immunity is generated by natural infection versus vaccination with replication-defective viral vectors. Neutralizing activity generated by natural infection is more determined by fiber-specific antibody, while rAd vector immunization elicited neutralizing activity is more dependent on hexon-specific antibody. Hence, evaluating the effect of pre-existing immunity is complex in subjects who had received at least one dose of rAd5 vaccine before as a minority had exposure to natural infection prior to receiving the initial rAd5 vaccine [18].

Prior to this study all rAd5 vaccinations in VRC clinical trials were administered by needle and syringe. This study was conducted to evaluate whether Biojector delivery of rAd5 would improve immunogenicity to the recombinant antigen as it does for DNA plasmid vaccines. We report here the results of a Phase I clinical trial comparing Biojector to N/S delivery of rAd5 vaccine in a healthy volunteer population divided into two groups comprised of those receiving a primary immunization (group 1) or those receiving a secondary immunization after having received at least one rAd5 HIV injection in a prior study (group 2).

## Methods

The protocol for the trial and supporting CONSORT checklist is provided as Checklist S1.

### Ethics Statement

The study was reviewed and approved by the National Institute of Allergy and Infectious Diseases Institutional Review Board and was performed in accordance with all applicable U.S. Food and Drug Administration regulations and principles expressed in the Declaration of Helsinski. All subjects gave written informed consent prior to study participation. The clinical trial reported here was submitted to clinicaltrials.gov on June 28, 2008 and is registered as NCT00709605.

### Objectives

The primary objective of this protocol was to characterize the safety, tolerability and immunogenicity profile of the HIV rAd5 vaccine comparing two different methods of intramuscular administration-needle and syringe versus needle-free pressure injection device (Biojector) in subjects receiving primary immunization and those who had previously received at least one injection of this study vaccine.

### Participants

The participants in this clinical trial were healthy, HIV-uninfected males and non-pregnant females between the ages of 18 and 55 at the time of enrollment. To address concerns raised by the Step Study, only subjects with low risk of HIV exposure were enrolled [19]. Low risk of HIV infection was defined as sexual abstinence, two or fewer mutually monogamous partners who were HIV uninfected with no history of drug use, or two or fewer partners who were HIV uninfected with no history of drug use and regular usage of barrier contraception.

### Study design and procedures

VRC 015, a Phase I open label clinical trial was conducted at the National Institutes of Health (NIH), Bethesda MD, by the VRC, National Institute of Allergy and Infectious Diseases (NIAID), NIH, Department of Health and Human Services (DHHS).

Vaccine-naïve subjects (group1) were randomized at screening, as defined by their adenovirus serotype-5 antibody (Ad5 Ab) titers, in a 1∶1 ratio to receive a single rAd5 vaccination intramuscularly (IM) by either Biojector or N/S. Group 1 included eight subjects with low (<1∶500) and twelve subjects with high (>1∶500) adenovirus serotype-5 antibody (Ad5 Ab) titers at screening equally distributed between the Biojector and N/S groups. Consideration of Ad5 Ab titer in the randomization ensured balance of Ad5 serostatus within each injection method to allow comparison of immune responses by injection method. Group 2 included participants who had received at least one rAd5 injection in a prior study. There were no limitations on the timing of their previous rAd5 dose and they were randomized in a 1∶1 ratio to receive vaccine via Biojector or N/S. Randomization to the injection device became known to both the study staff and the subjects by completion of an enrollment in the electronic database.

All subjects self-reported reactogenicity on 5-day diary cards following the study injection. Local reactogenicity parameters solicited on the diary card included sore/blister at site of vaccination, pain/tenderness, swelling and redness. Solicited parameters of systemic reactogenicity were malaise, myalgia, headache, chills, nausea and highest measured temperature. Reactogenicity was graded as mild, moderate, or severe. Clinical and laboratory follow up was through week 24 of the study. All adverse events (AE's) were reported for the entire duration of the study for individual participants, assessed for relationship to study vaccine (unrelated, probably not/unlikely, possibly, probably or definitely related). The Division of AIDS table for Grading Adult and Pediatric Adverse Events, December 2004 was used for severity grading of adverse events. To prepare a summary of data, AE's were coded using the Medical Dictionary for Regulatory activities (MedDRA).

### Vaccine

The study vaccine, developed by the VRC, NIAID, NIH was VRC-HIVADV014-00-VP and is a replication deficient combination vaccine containing four recombinant adenoviral serotype 5 vectors that code for HIV-1 Gag/Pol polyproteins from clade B and Env glycoprotein from clade A, clade B and clade C combined in a 3∶1∶1∶1 ratio respectively in a final formulation buffer (FFB) [20], [21].

### Enzyme-linked immunospot assays (ELISpot)

The frequency of antigen/vaccine specific T cells was determined as previously described [7]. Cryopreserved PBMCs were stimulated overnight by peptide pools representing the individual vaccine antigens. IFN-γ ELISpot was performed using a commercial kit (MABTECH), read on a CTL ELISpot image analyzer (Cellular Technology Ltd; Cleveland, OH), and expressed as spot forming cells (SFC) per million PBMC.

### Flow cytometric analysis and intracellular cytokine staining (ICS)

Cryopreserved PBMCs were stimulated by peptide pools for 6 hours with brefeldin A. Permeabilized fixed cells were evaluated by flow cytometry for expression of CD3, CD8, CD4, and IFN-γ, TNF-α, and/or IL-2, then analyzed using FlowJo software (TreeStar; Ashland, OR) as previously described [7].

### Measurement of antibody responses

Standard ELISAs were utilized to determine antibody responses to viral antigens encoded within the vaccine. End-point titers of antibodies were determined using 96-well Immulon2 (Dynex Technologies) plates coated with a preparation of purified recombinant HIV proteins derived from the same sequences as the vaccine antigens [7]. End-point titer was calculated as the most dilute serum concentration that gave an optical density reading of >0.2 above background.

### Peptides

Peptides (15-mers overlapping by 11) matching the sequences of the HIV-specific antigens expressed by the vaccines were used at >80% purity. They were pooled according to antigen (EnvA, EnvB, EnvC, Nef Gag and Pol) and were used at a final concentration of 2.5 µg/ml to stimulate vaccine induced T cells *in-vitro*.

### HIV-1 Diagnostic Testing

The Ortho VITROS HIV-1/HIV-2 EIA kit was used for diagnostic testing. For reactive results, Western blot analyses were done at Mayo Laboratory using the GS HIV-1 Western Blot (BioRad Laboratories, Redmond, WA). The AMPLICOR HIV-1 MONITOR Test ver.1.5 (Roche Molecular Systems, Indianapolis, IN) was used till November 2009 after which the COBAS AmpliPrep HIV-1 Test, version 2.0 (Roche Molecular Systems, Indianapolis, IN) was used for HIV RNA PCR testing regardless of EIA result at all testing time points.

### Data analysis and statistics

The statistical analysis of results for this study is consistent with the protocol plan and the methods published previously in Phase 1 studies performed with VRC HIV vaccines [10], [21], [22]. Descriptive statistics are applied to the safety data. For the T-cell responses as measured by the ELIspot assay the positivity criteria have been established and are defined by both a statistical test and a minimum magnitude threshold. The threshold was pre-specified for each antigen and determined by an assay validation process. A positive response for the ELISA assay is defined by an end-point titer ≥30. Comparisons for frequency of response were performed with Fisher's Exact Test and for magnitude of response with Wilcoxon Rank sum test.

## Results

### Study conduct and population

**Figure 1:**
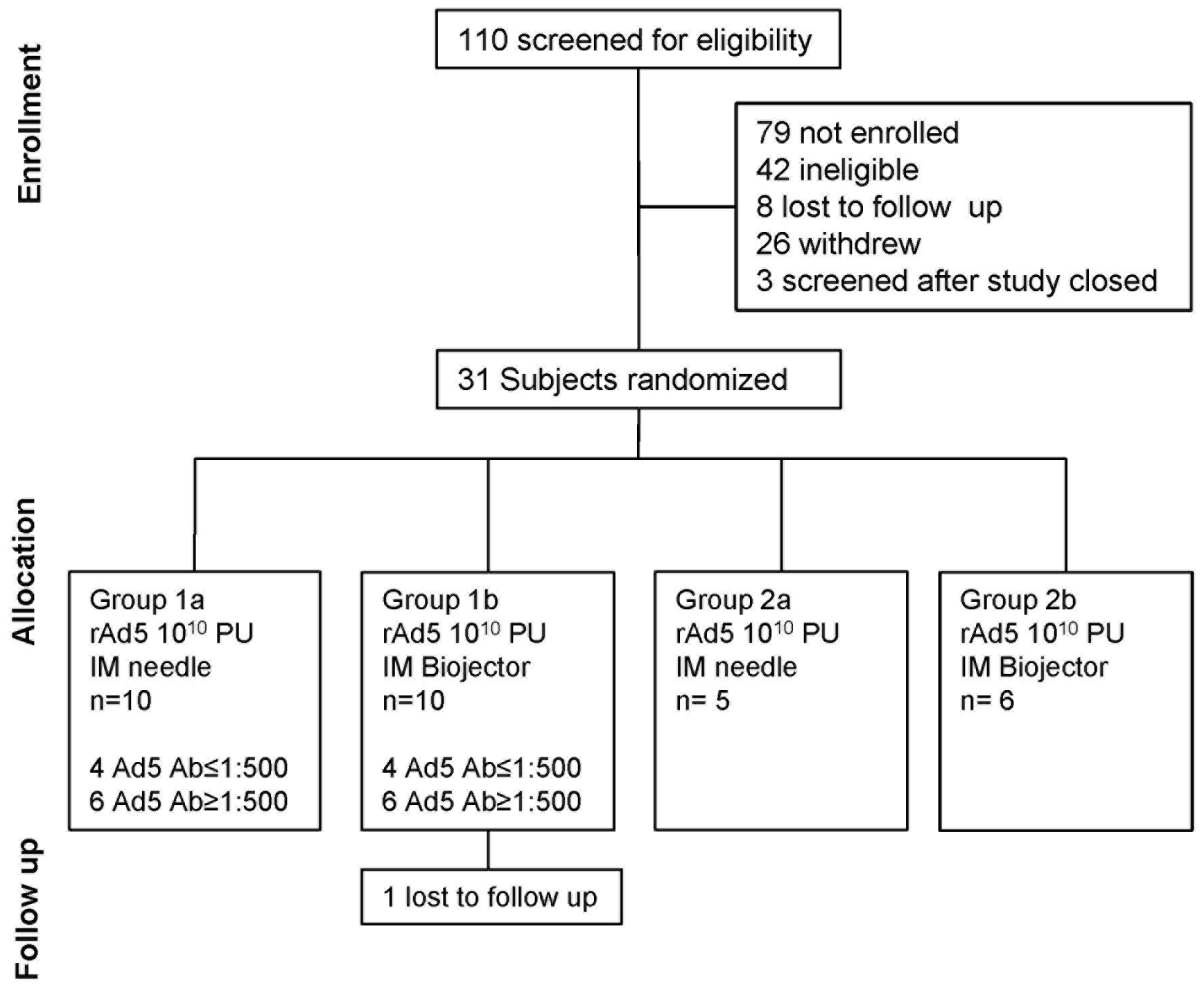
Schematic diagram of study design and vaccination schedule. The CONSORT diagram indicates the number of subjects screened to complete enrollment of 31 subjects. Subjects were stratified as group 1 (primary immunization) versus group 2 (secondary immunization) and randomized to receive rAd5 vaccine by Biojector or needle and syringe. Group 1 included 8 subjects with adenovirus serotype 5 neutralizing antibody reciprocal titer ≤500 randomized equally into sub groups 1a and 1b.

A flow diagram of the trial is shown in Figure 1. A total of 110 subjects were screened between September 4, 2008 and December 17, 2009 and of those thirty-one subjects were enrolled between February 18, 2009 and January 12, 2010. The final study injection was administered in January 12, 2010 and the last required clinic visit was completed in June 29, 2010. Participant demographic characteristics are shown in Table 1. The mean age of the participants was 33.1 years and 51.6% were male. The majority of the participants were white and there were no significant differences in age or gender by study group. A balance of subjects was attained by enrolling at least 8 subjects with low and 8 subjects with high pre-existing Ad5 Ab titers equally divided between the Biojector and N/S subgroups in group 1. Ad5 Ab titer was not considered in the randomization plan for group 2.

**Table 1:** Baseline Demographics.

Category	Characteristic	Vaccine Naïve Needle (N = 10)	Vaccine Naïve Biojector (N = 10)	Rollover Enrollments Needle (N = 5)	Rollover Enrollments Biojector (N = 6)	VRC 015 Overall (N = 31)
GENDER	Male – no. (%)	7 (70)	3 (30)	3 (60)	3 (50)	16 (51.6)
	Female – no. (%)	3 (30)	7 (70)	2 (40)	3 (50)	15 (48.4)
AGE	Mean [S.D.]	29.7 [6.0]	33.9 [9.7]	36.2 [6.3]	35.0 [8.5]	33.1 [7.9]
	Range	[23, 43]	[20, 50]	[28, 45]	[24, 46]	[20, 50]
RACE	Asian– no. (%)	0 (0)	0 (0)	0 (0)	1 (16.7)	1 (3.2)
	Black or African American– no. (%)	1 (10)	3 (30)	0 (0)	0 (0)	4 (12.9)
	White– no. (%)	9 (90)	5 (50)	5 (100)	5 (83.3)	24 (77.4)
	Multiracial– no. (%)	0 (0)	2 (20)	0 (0)	0 (0)	2 (6.5)
ETHNICITY	Non-Hispanic/Latino– no. (%)	8 (80)	9 (90)	4 (80)	5 (83.3)	26 (83.9)
	Hispanic/Latino– no. (%)	2 (20)	1 (10)	1 (20)	1 (16.7)	5 (16.1)
BMI	Mean [S.D.]	24.7 (3.0)	27.1 (4.5)	25.9 (4.1)	25.9 (5.2)	25.9 (4.0)
	Range	[18.5, 28.7]	[19.7, 33.4]	[21.5, 31.4]	[19.0, 32.4]	[18.5, 33.4]
EDUCATION	High school graduate/GED– no. (%)	2 (20)	1 (10)	1 (20)	1 (16.7)	5 (16.1)
	College/University– no. (%)	5 (50)	9 (90)	1 (20)	4 (66.7)	19 (61.3)
	Advanced degree– no. (%)	3 (30)	0 (0)	3 (60)	1 (16.7)	7 (22.6)

Group 1 included eight subjects with low (<1∶500) and twelve subjects with high (>1∶500) Ad5 Ab titers at screening. The 8 subjects with low Ad5 Ab titers included 3 with undetectable (<12) Ad5 neutralizing titers at baseline and 5 with relatively low titers (<1∶50). Group 1 subjects with high (≥1: 500) Ad5 Ab included 11 with reciprocal titers >1000 and one with a reciprocal titer of 754. Enrollment and follow up of subjects is outlined in Figure 1. All study participants completed their study vaccination visits and 30 of 31 participants completed the protocol through the planned clinical follow up for 24 weeks. One subject from group 1 was lost to follow up after completion of rAd5 vaccination and seven day follow up visit (refer to Figure 1). Group 2 included eleven subjects from prior rAd5 vaccine studies; six had received two prior rAd5 vaccine injections and five had received one prior injection. In addition, four of the subjects were Ad5 Ab seropositive prior to the receipt of their first rAd5 vaccination.

### Vaccine safety

**Figure 2:**
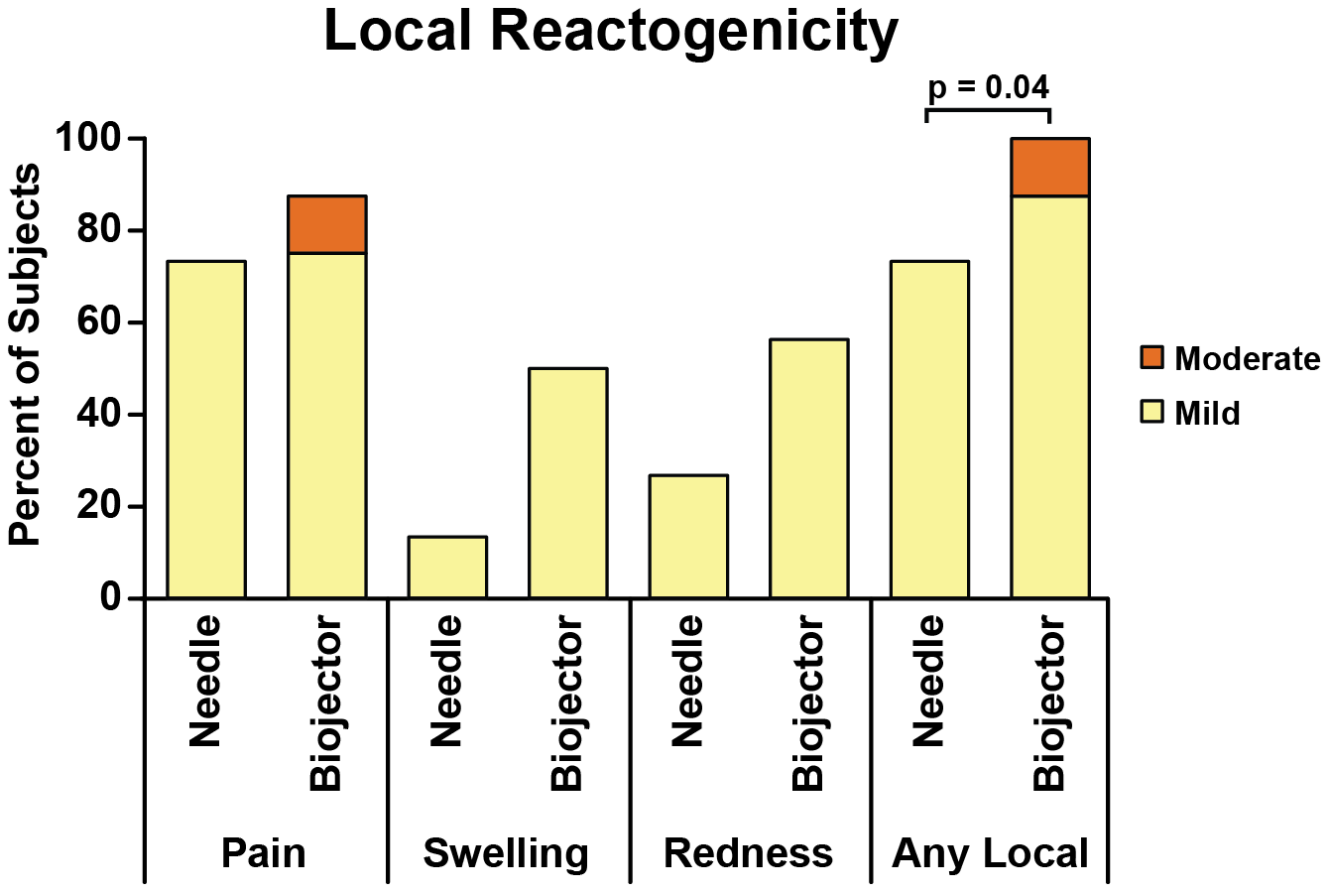
Local reactogenicity following rAd5 vaccine. All subjects who received rAd5 vaccination by needle injection (n = 15) or by Biojector injection (n = 16) were counted once at worst severity over 5 days for solicited local parameters. The graph shows the percentage with mild or moderate reaction; there were no severe reactions. The results for local parameters of pain, swelling and redness are shown individually, while “Any Local” represents the worst severity for any of the local parameters. There were no statistically significant differences between the needle and Biojector reactogenicity except for “Any Local”.

**Figure 3:**
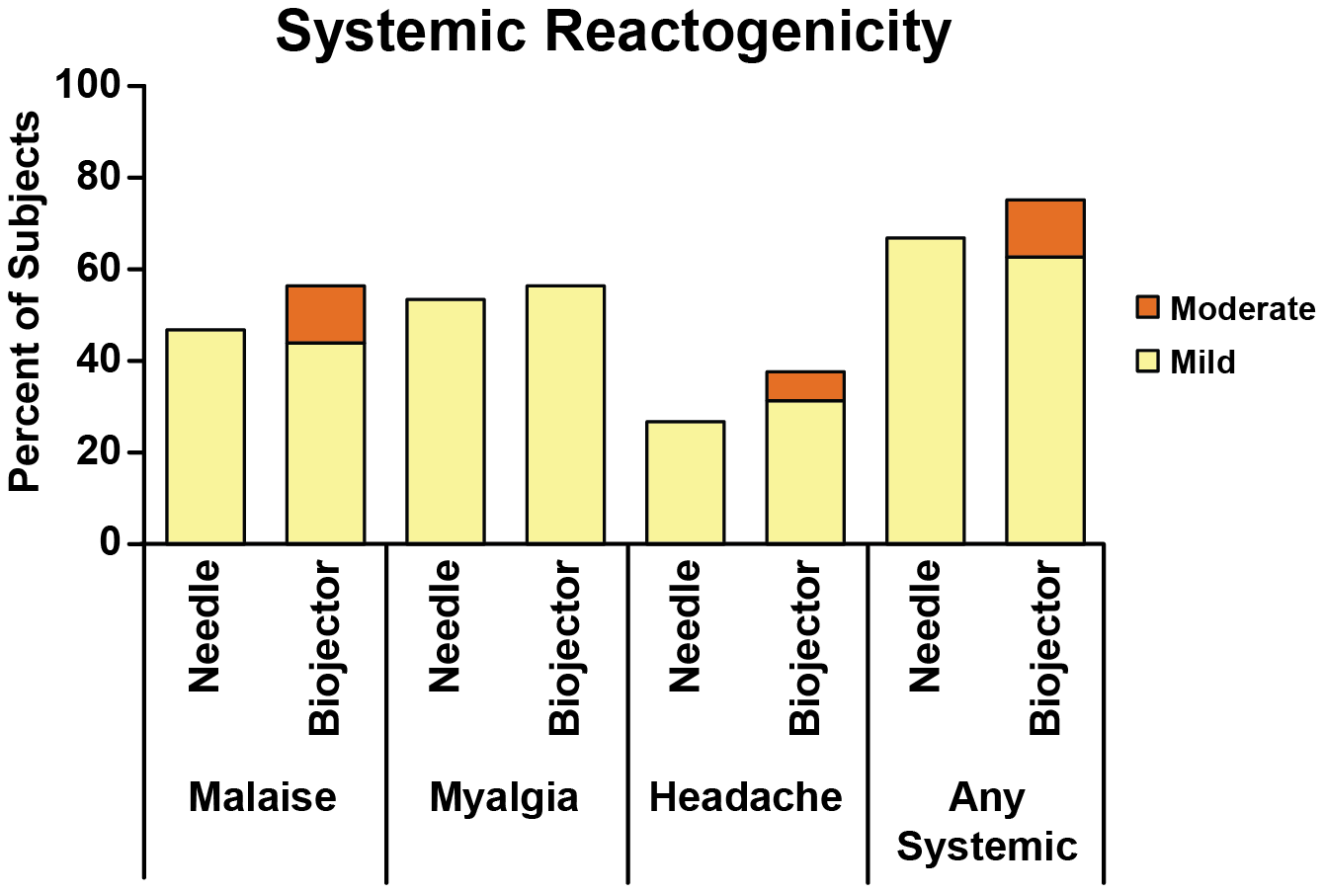
Systemic reactogenicity following rAd5 vaccine. All subjects who received rAd5 vaccination by needle injection (n = 15) or by Biojector injection (n = 16) were counted once at worst severity over 5 days for solicited systemic parameters. The results for systemic parameters of malaise, myalgia and headache are shown, while solicited parameters chills, nausea and fever, which were reported in only 1 or 2 subjects each, are not shown. “Any Systemic” represents the worst severity for any of the systemic parameters.

Overall, the administration of rAd5 vaccine was well tolerated in all 31 subjects. The frequency of local reactogenicty was 100% for the Biojector groups versus 73% for the N/S groups (p = 0.04 by Fisher's Exact Test). In the Biojector groups, 14/16 (87.5%) experienced mild reactogenicity and 2/16 (12.5%) reported moderate pain and tenderness 1–2 days post vaccination. Of those reporting reactogenicity in the N/S groups, all reported mild reactogenicity. Systemic reactogenicity was seen in 12/16 (75%) in the Biojector groups and 10/15 (66.7%) in the N/S groups (p = 0.7). Of those reporting reactogenicity in the N/S groups all reported mild reactogenicity. Among subjects in Biojector groups 10/16 (62.5%) reported mild reactogenicity and 2/16 (12.5%) reported moderate reactogenicity (malaise and headache). The overall reactogenicity of rAd5 vaccine by Biojector compared to N/S delivery is shown in Figures 2 and 3.

Preexisting Ad5 Ab titer did not appear to affect reactogenicity and the incidence of reactogenicity was similar between subjects from group 1 with low Ad5 and high Ad5 Ab titers regardless of the method of vaccine delivery as also seen previously in VRC 008 study and other clinical trials [3], [23]. The rate of local reactogenicity was 6/8 (75%) in low Ad5 Ab titer groups and 10/12 (83.3%) in high Ad5 Ab titer subjects. Mild or moderate systemic reactogenicity was noted in 5/8 (62.5%) in the low Ad5-Ab titer versus 9/12 (75%) in the high Ad5 Ab titer subjects. The reactogencity events resolved without sequalae and were consistent with earlier phase I experience with recombinant adenoviral vectors [21].

There were no serious adverse events. After the administration of the rAd5 vaccine, three mild adverse events were assessed as definitely related to study vaccine: two subjects in group 1 had a superficial skin erosion/scab formation (maximum diameter 0.5 cm) at the site of injection at 5 days post administration by Biojector and one subject in group 1 had erythema and induration at the injection site that occurred 10 days post injection by Biojector. Six subjects experienced bruising at the injection site 1–6 days following vaccination, which were assessed as related to the use of device and not to the study vaccine. Of the six subjects, 4 received vaccination by Biojector and 2 subjects via N/S. All the unsolicited adverse events were mild or moderate.

### Vaccine specific antibody responses

Antibody responses measured by ELISA showed similar responses against EnvA, EnvB and EnvC subtypes. The frequency and level of responses were similar for the Biojector and N/S delivery sub groups. In group 1, 6 of 10 subjects had positive Env-specific antibody titers to EnvA (median 90, range 30-810) in the N/S delivery group and 6 of 9 subjects had positive responses (median 30, range 30-270) in the Biojector group (where positive responses were defined as end point titers of ≥30). All group 2 subjects had Env-specific antibody titers to EnvA and the magnitude of responses were similar in the N/S (median 2430, range 810-7290) and Biojector groups (median 4860, range 270-7290). Biojector delivery of rAd5 vaccine did not improve Env-specific antibody titers in primary immunization or secondary immunization relative to delivery by needle and syringe.

**Figure 4:**
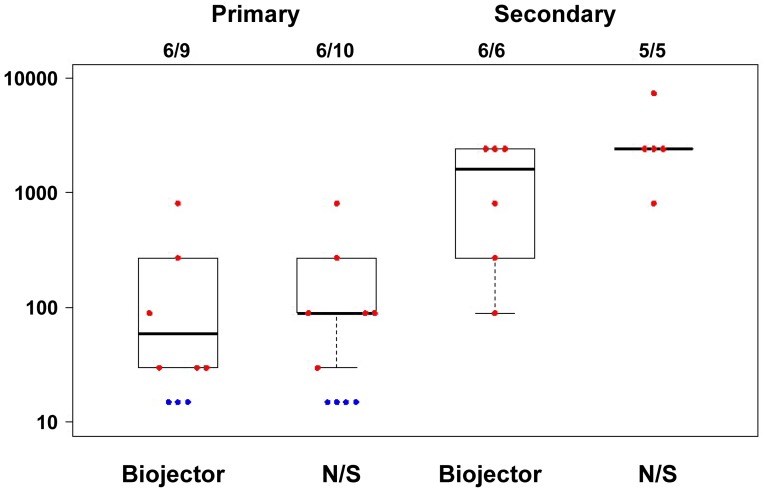
Env A-specific antibody response is higher in secondary immunization. Antibody responses measured by ELISA against HIV-1 envelope proteins matching the vaccine antigen. Data is shown from 4 weeks post rAd5 vaccine for both primary (group 1) and secondary immunization (group 2) subjects by route of administration as individual data points and box plots showing the median, 25^th^ and 75^th^ quartiles. Red represents the responders and blue represents the non-responders. Anti-EnvA antibody levels are plotted as log 10 of ELISA titer. These are representative of similar responses to EnvB and EnvC.

All 11 group 2 subjects had EnvA-specific antibody responses as compared to 12 of 19 group 1 subjects. Among these responders, the response magnitudes in group 2 were more than 10-fold higher (median 2430 EnvA, range 270-7290) 4 weeks post rAd5 vaccine than in group 1 (median 90, EnvA, range 30-810) as seen by medians plotted in Figure 4. A booster dose of rAd5 induced more than10-fold higher responses in those that received secondary immunization.

As mentioned earlier, group 2 comprised of subjects who had received their previous dose of rAd5 at varying intervals related to their past study participation. The range of intervals varied from 462 days (66 weeks) to 1772 days (253 weeks). In this study we observed comparable boosting of Env-specific antibody titers in subjects following a secondary homologous rAd5 vaccination, regardless of interval between prime and boost.

**Figure 5:**
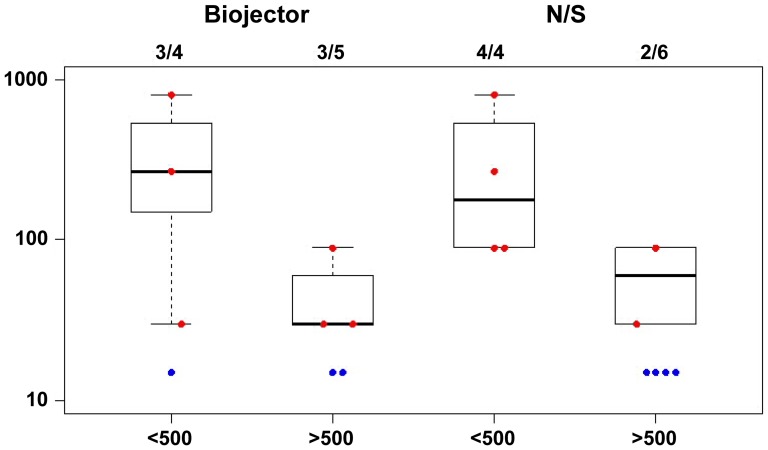
Group 1 Env-specific antibody responses stratified by pre-existing reciprocal 90% Ad5 neutralization titers. Antibody responses were measured using ELISA against HIV-1 envelope proteins matching vaccine antigen. Data from 4 weeks post rAd5 vaccine is shown as individual data points and box plots showing the median, 25^th^ and 75^th^ quartiles for group 1 stratified by reciprocal dilution 90% Ad5 neutralization titer. A comparison of Biojector and N/S mode of delivery is shown. Red represents the responders and blue represents the non-responders. Anti-EnvA binding antibody levels are plotted as log 10 of ELISA titer, and are shown as representative of responses to EnvB and EnvC.

To evaluate whether pre-existing Ad5 vector immunity influenced immune responses in those receiving primary immunization, group 1 responses were stratified by pre-existing reciprocal dilution 90% Ad5 Ab neutralization titers in Figure 5. Responses were modestly higher in those with low pre-existing Ad5 Ab (≤1: 500) (p = 0.002 by Wilcoxon Rank Sum Test) as previously reported [7], [24], and the mode of delivery did not affect response. The baseline Ad5 Ab titer was not considered in the overall study schema for group 2 which included only a small number of subjects in each Ad5 Ab titer strata. Env-specific ELISA responses in group 2 subjects were approximately 10 fold higher regardless of baseline Ad5 Ab titer at start of study. (data not shown)

### Vaccine induced T cell responses

**Figure 6:**
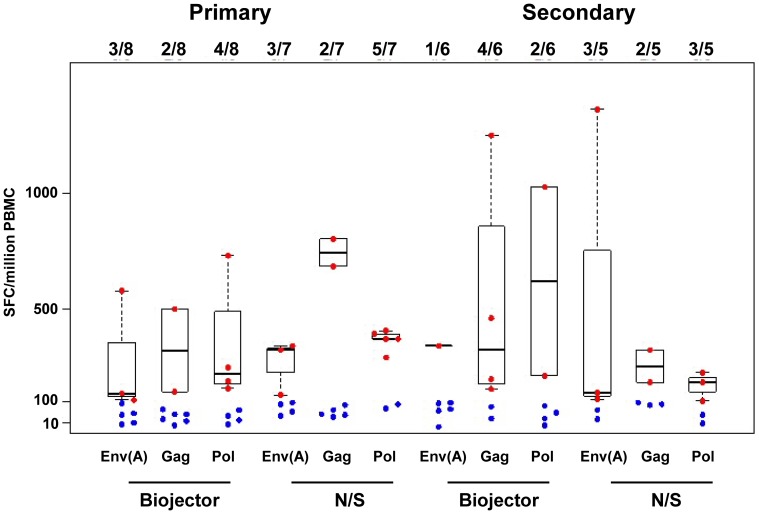
Interferon-γ ELISpot responses to EnvA, Gag & Pol by group. The box plots represent a comparison of the median magnitude, 25^th^ and 75^th^ quartiles for IFN-γ ELISpot responses [spot-forming cells per million peripheral blood mononuclear cells (PBMC)] to EnvA, Gag and Pol matched peptide stimulation by group. Data from 4 weeks post rAd5 vaccine is shown for mode of delivery via Biojector and N/S. Red represents the responders and blue represents the non-responders. Similar responses were noted for EnvB and EnvC.

The frequency of IFN-γ ELISpot responses to EnvA, Gag and Pol peptides by group and method of vaccine delivery are shown in Figure 6. Vaccine elicited HIV-1 specific T cell responses were detected at 4 weeks after the rAd5 vaccine, (the primary end point) and also at weeks 12, 24, 76, and 128 after vaccination. IFN-γ ELISpot and ICS CD4+ and CD8+ T cell assays were completed for 15/20 subjects in group 1 and all subjects in group 2 at the 4 weeks post rAd5 time point. Five subjects did not have complete T cell analyses, due to insufficient cells available for analysis (3 subjects from group 1a and 2 subjects from group 1b). The frequency of IFN-γ ELISpot HIV-1 specific responses for any peptide pool at 4 weeks post rAd5 was 6/7 (87.5%) for N/S and 5/8 (62.5%) in Biojector groups in group 1. In group 2, the frequency of IFN-γ ELISpot responses for any peptide pool at 4 weeks post rAd5 was 5/5 (100%) in N/S and 5/6 (83.3%) in the Biojector sub groups. The specificity of the ELISpot responses were focused towards Env, as noted in prior studies.

The cumulative median ELISpot responses to matched peptides (sum of Gag, Pol, Nef and highest Env responses) were similar in group 1 and group 2 subjects. Among responders, group 1 primary immunization subjects had modestly higher cumulative ELISpot responses in N/S group (median = 717) as compared to Biojector group (median = 503). Similarly, in group 2, cumulative ELISpot responses were modestly higher with N/S (median = 597) than Biojector delivery (median = 363). There was no statistically significant difference in magnitude of ELISpot responses based on mode of delivery (p = 0.43 for group 1, p = 0.55 for group 2 by Wilcoxon Rank Sum test).

**Figure 7:**
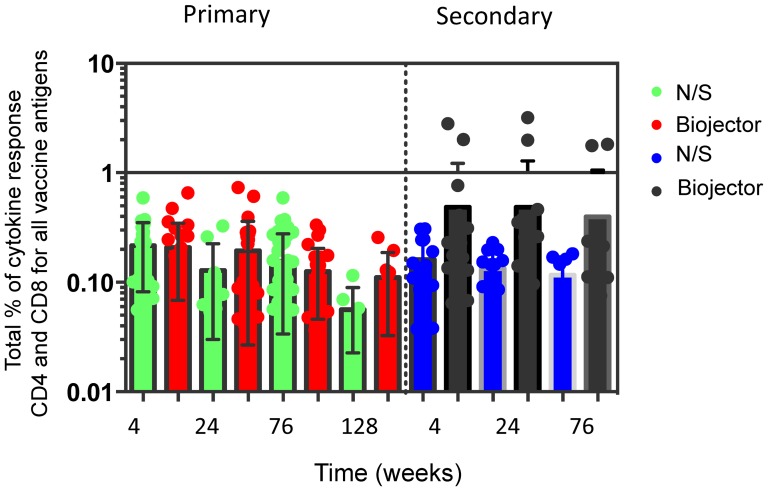
Total CD4 and CD8 T-cell responses (IFN-γ, IL-2 and TNF-α) to all vaccine antigens (Gag, Pol and Env) were not significantly (p = >0.05) improved by Biojector in secondary immunization. This graph represents total CD4 and CD8 T cell responses to all vaccine antigens Env, Gag and Pol for each study sub group. Total percentage of cytokine responses on log scale is shown on the y-axis. The combined T cell responses are shown over a longitudinal period at 4, 24 and 76 and 128 weeks following rAd5 vaccine on the x-axis. Responses are shown for the Biojector and needle groups for both primary and secondary immunization.

Using the ICS assays, CD4+ and CD8+ T cell responses were measured at 4 weeks post rAd5 vaccination and through week 24. There were consistently sustained low frequencies of CD4 and CD8 vaccine antigen-specific T cell responses detected by ICS by either mode of vaccine delivery in both group 1 primary immunization and group 2 secondary immunization subjects. Analysis of the total (IFN-γ, IL-2 and TNF-α) responses demonstrated that few subjects made consistently high frequency cytokine responses despite an indication that secondary immunization with Biojector elicited higher cytokine responses (especially to HIV-Env A and Pol peptides), overall this was not significant (Figure 7).

## Discussion

The use of needle-free injection systems is an alternative to N/S administration of vaccines and has potential safety advantages by eliminating the risk of blood borne pathogen transmission. Moreover, previous clinical studies have shown improved immune responses as a result of Biojector delivery of vaccines [1], [2], [25]. In particular, DNA vaccine delivery by Biojector was associated with improved antibody and T cell responses following rAd5 vaccine boost [3]. The current study was conducted to evaluate if using this method of delivery of rAd5 would be safe and immunogenic in subjects receiving primary and secondary immunization. We found that overall; rAd5 vaccination by this mode of delivery was well tolerated. Needle-free delivery of rAd5 was immunogenic indicating the integrity of the rAd5 vector is not compromised by shear forces associated with this mode of delivery. However needle-free injection of rAd5 vaccine does not increase its immunogenicity as previously observed for DNA vaccines. This suggests that needle-free delivery may have increased the immunogenicity of DNA by improving transduction efficiency, but for a vector like rAd5, which already has high transduction efficiency, the added value appears to be limited.

Needle-free injection systems like the Biojector device eject fluid through a small orifice under pressure penetrating all layers of skin. This potentially creates a more widespread intramuscular distribution, which improves immunogenicity possibly due to wider dispersion and activation of dendritic cells in the skin [26]. While needle-free delivery of rAd5 vaccine showed an increased frequency of local and systemic reactogenicity in the Biojector groups in comparison to the N/S groups, this observation would not preclude clinical use of this device. Increased reactogenicity was also noted previously in VRC 008 study comparing Biojector and N/S delivery of a HIV DNA vaccine. This finding is consistent with previous experience with needle free devices used for vaccine delivery [27], [28].

Data from non-U.S. populations have shown as high as 90% of Ad5 seroprevlance in populations targeted for vaccination especially in the developing world [29]. Pre-existing adenovirus immunity has been reported to diminish the magnitude of immune responses induced by the Ad5 vector [30]. In the RV172 trial there was a non-significant trend of lower magnitude of IFN-γ ELISpot responses in participants with higher baseline Ad5 titers. A modest reduction in the frequency of IFN-γ ELISpot, CD4+ and CD8+ T cell ICS responses was also seen in the Ad5 seropositive vaccine recipients compared to those who were seronegative in the HVTN 204 study [31]. Other mechanisms of delivery may potentially modulate the effect of anti-vector immunity upon the immunogenicity of the rAd5. In parallel with the current study, another study compared subcutaneous, intradermal and intramuscular routes of administration of rAd5 vaccine boost after a DNA HIV vaccine prime and revealed a higher frequency of local reactions after subcutaneous and intradermal administration but no difference in humoral or cellular responses in the three study arms [32]. In our study, Biojector delivery did not significantly improve vaccine immunogenicity in the setting of pre-existing Ad5 Ab.

Our study found that Env-specific antibody responses were more than 10-fold higher in subjects who received a secondary dose of rAd5 vaccine (group 2) than after a primary dose (group 1). A similar finding was noted in the HVTN 068 study where homologous immunization 24 weeks after the initial dose with a recombinant adenoviral vector vaccine enhanced post-boost Env specific antibody responses (20-fold to gp 140 and 8-fold to gp 41) in subjects with no pre-existing Ad5 immunity, but no increase in T cell responses were seen [17]. Group 2 included a minority of subjects (one in the N/S and 3 in the Biojector groups) who had preexisting immunity to Ad5 as a result of natural infection prior to receiving their first dose of rAd5 vaccine. The boost in response observed after another dose of rAd5 vaccine was seen in subjects with pre-existing neutralizing activity derived from natural infection and rAd5 immunization as well as in those with neutralizing activity from prior rAd5 immunizations alone. It is possible that the peak antibody response could be slightly different in the primary versus the secondary immunization groups. Therefore, comparing the responses at 4 weeks post immunization could potentially be misleading. However, in prior studies, the differences in 2, 4, or 6 weeks time points post immunization have been subtle and would not account for a 10-fold difference in response.

Subjects receiving secondary immunization included in our study had received their previous dose of rAd5 at varying intervals in the past with wide range of time periods between their previous and current rAd5 doses. We found that homologous boosting with rAd5 gene-based vectors can boost antibody responses despite pre-existing vector-specific immunity and this effect can be seen regardless of a prolonged interval between prime and boost.

IFN-γ ELISpot responses were similar in subjects receiving primary or secondary immunization. CD4+ and CD8+ T cell responses evaluated at 4 weeks post rAd5 vaccine were of low magnitude and remained low throughout the study. Previous reports have described that rAd5 HIV vaccine induces primarily CD8+ T cell responses, which are considered an important element in controlling HIV infection [33]–[35]. There may be a modest increase in CD4+ and CD8+ T cell responses in those receiving secondary immunization with Biojector delivery but the group sizes were too small to achieve statistical significance.

Although our study was limited by small group sizes and varying intervals of the previous rAd5 vaccine dose in those receiving a secondary immunization, we showed that Biojector delivery of the VRC rAd5 vaccine is well tolerated. Although needle-free delivery of rAd5 induced significant humoral and cellular immune responses it does not significantly improve vaccine immunogenicity. The most important finding in this trial was that homologous boosting with rAd5 gene-based vectors boosts antibody responses despite pre-existing vector-specific immunity and regardless of interval between prime and boost. Even though the negative results of HVTN 505 study will halt further evaluation of this rAd5 vaccine, results from our study remain applicable to other vaccine platforms that utilize recombinant adenoviral vectors. Our study suggests whether delivered needle-free or by N/S, additional doses could be considered if needed to extend the duration of immunity in prior recipients of recombinant adenoviral vector vaccines.

## Supporting Information

Checklist S1 VRC 015 study CONSORT checklist of information.
(DOCX)

Protocol S1 Study Protocol VRC 015, A phase I, open-label clinical trial to evaluate the safety, tolerability and Immunogenicity of a multiclade recombinant HIV-1 adenoviral vector vaccine in uninfected adults randomized to needle or Biojector methods of intramuscular injection.
(PDF)
