# Supplementary material for: Homologous Boosting with Adenoviral Serotype 5 HIV Vaccine (rAd5) Vector Can Boost Antibody Responses despite Preexisting Vector-Specific Immunity in a Randomized Phase I Clinical Trial
Source: PLoS One. 2014 Sep 29;9(9):e106240. doi: 10.1371/journal.pone.0106240 (PMC4179264; doi:10.1371/journal.pone.0106240)
Supplement: Protocol S1 — Study Protocol VRC 015, A phase I, open-label clinical trial to evaluate the safety, tolerability and Immunogenicity of a multiclade recombinant HIV-1 adenoviral vector vaccine in uninfected adults randomized to needle or Biojector methods of intramuscular injection. (PDF) [file pone.0106240.s002.pdf]

Version 5.0
January 10, 2012

VACCINE RESEARCH CENTER

Protocol VRC 015
(NIH 08-I-0171)
(DAIDS-ES ID 10642)

A Phase I, Open-Label Clinical Trial to Evaluate the Safety, Tolerability and Immunogenicity of a Multiclade Recombinant HIV-1 Adenoviral Vector Vaccine, VRC-HIVADV014-00-VP, in Uninfected Adults Randomized to Needle or Biojector Methods of Intramuscular Injection

Vaccine Provided by
Vaccine Research Center/NIAID/NIH, Bethesda, MD

Clinical Trial Sponsored by:
National Institute of Allergy and Infectious Diseases (NIAID)
Vaccine Research Center (VRC)
Bethesda, Maryland

IND Sponsored by:
National Institute of Allergy and Infectious Diseases
Division of AIDS (DAIDS)
Bethesda, Maryland

BB-IND 11661 - *held by DAIDS*

Principal Investigator:
Barney S. Graham, M.D., Ph.D.
Vaccine Research Center, National Institute of Allergy and Infectious Diseases (NIAID)
National Institutes of Health (NIH)
Bethesda, MD 20892

IRB Initial Review Date: June 2, 2008

TABLE OF CONTENTS

Page
Abbreviations Used in VRC 015 5
Table of IND and Protocol Numbers Discussed in VRC 015 7
Précis....8

1. INTRODUCTION AND RATIONALE.... 10

1.1 HIV-1: Etiology, Disease Course, and Epidemiology.... 10
1.2 Previous Experience with the Study Vaccine, VRC-HIVADV014-00-VP.... 10
1.3 Previous Experience with Other rAd5 HIV Vaccines.... 11
1.4 Rationale for Evaluating Biojector Administration of rAd5 Vaccine.... 12
1.5 Measures of Immunogenicity.... 15

2. BACKGROUND ON VACCINE.... 16

2.1 Adenoviral Vectors in VRC-HIVADV014-00-VP.... 16
2.2 Manufacture of the Final Vaccine Product.... 17

3. STUDY OBJECTIVES.... 18

3.1 Primary Objectives.... 18
3.2 Secondary Objectives.... 18
3.3 Exploratory Objectives.... 18

4. STUDY DESIGN.... 18

4.1 Study Population.... 19
4.1.1 Inclusion Criteria.... 20
4.1.2 Exclusion Criteria.... 22
4.2 Clinical Procedures and Laboratory Assays.... 24
4.2.1 Screening.... 24
4.2.2 Day 0 through Week 24.... 24
4.2.3 Long-term Follow-up:.... 27
4.3 Monitoring for HIV Infection.... 27
4.4 Intercurrent HIV Infection.... 28
4.5 Concomitant Medications.... 28
4.6 Criteria for Withdrawal of a Subject from Study Participation.... 28
4.7 Criteria for Pausing the Study and Resuming the Study.... 28

5. SAFETY AND ADVERSE EVENT REPORTING.... 29

5.1 Adverse Events.... 29
5.2 Serious Adverse Events (SAE).... 29
5.3 Adverse Event Reporting to the IND Sponsor.... 30
5.3.1 Reporting to the IND Sponsor through Study Week 24.... 30
5.3.2 Reporting to the IND Sponsor During Long-term Follow-up.... 31
5.3.3 Attribution Categories.... 32
5.4 Reporting to the Institutional Review Board.... 33
5.4.1 Adverse Event Reporting to the NIAID IRB.... 33
5.4.2 Unanticipated Problem Reporting to the NIAID IRB.... 33
5.4.3 Protocol Violation Reporting to the NIAID IRB.... 34

5.5 Serious Adverse Event Reporting to the Institutional Biosafety Committee 34

6. STATISTICAL CONSIDERATIONS 34

6.1 Overview 34
6.2 Objectives 34
6.2.1 Safety Endpoints 34
6.2.2 Immunogenicity Endpoints 35
6.3 Sample Size and Accrual 35
6.3.1 Randomization of Treatment Assignments 35
6.3.2 Sample Size Considerations for Safety 36
6.3.3 Sample Size Considerations for Immunogenicity 37
6.4 Statistical Analysis 37
6.4.1 Analysis Variables 37
6.4.2 Baseline Demographics 37
6.4.3 Safety Analysis 38
6.4.4 Immunogenicity Analysis 38
6.4.5 Interim Analyses 39

7. PHARMACY PROCEDURES 39

7.1 Study Products and Injection Regimen 39
7.2 Study Agent Storage, Shipping and Presentation 39
7.2.1 Study Agent Storage 39
7.2.2 VRC-HIVADV014-00-VP Adenoviral Vector Vaccine, rAd5 39
7.3 Preparation of Study Agent for Administration 40
7.3.1 VRC-HIVADV014-00-VP Adenoviral Vector Vaccine: Preparation for Administration 40
7.3.2 Administration of VRC-HIVADV014-00-VP by Needle and Syringe 40
7.3.3 Administration of VRC-HIVADV014-00-VP by Biojector 40
7.4 Study Agent Labeling 41
7.5 Study Agent Accountability 41
7.6 Study Agent Disposition 41

8. HUMAN SUBJECT PROTECTIONS AND ETHICAL OBLIGATIONS 41

8.1 Informed Consent 41
8.2 Risks and Benefits 41
8.2.1 Risks 41
8.2.2 Benefits 43
8.3 Institutional Review Board 43
8.4 Protocol Registration 43
8.5 Subject Confidentiality 43
8.6 Plan for Use and Storage of Biological Samples 43
8.7 Subject Identification and Enrollment of Study Participants 44
8.7.1 Participation of Children 45
8.8 Compensation 45
8.9 Safety Monitoring 45

9. ADMINISTRATIVE AND LEGAL OBLIGATIONS 45

9.1 Protocol Amendments and Study Termination 45
9.2 Study Documentation and Storage 46
9.3 Study Monitoring, Data Collection and Data Monitoring 46
9.3.1 Study Monitoring 46
9.3.2 Data Collection 47
9.4 Language 47
9.5 Policy Regarding Research-Related Injuries 47

10. REFERENCES 48

APPENDICES

I Study Informed Consent Form 50
II Contact Information 65
III Schedule of Evaluations 67
IV Table for Grading Severity of Adverse Events 69

Abbreviations Used in VRC 015

Abbreviation	Term
AAE	acquired angioedema
ADL	activities of daily living
AE	adverse event
AIDS	Acquired Immunodeficiency Syndrome
ALT	alanine aminotransferase
AoU	Assessment of Understanding
β-HCG	human chorionic gonadotropin
Biojector	Biojector®2000
BMI	body mass index
CAB	Community Advisory Board
CAVE	Capital Area Vaccine Effort
CBC	complete blood count
cDNA	complementary deoxyribonucleic acid
cGMP	current Good Manufacturing Practices
CMV	Cytomegalovirus
CsCl	cesium chloride
CTL	cytotoxic T lymphocytes
DAIDS	Division of AIDS
DNA	deoxyribonucleic acid
EAE	expedited adverse event
ELISA	enzyme-linked immunosorbent assay
ELISpot	enzyme-linked immunospot assay
Env	Envelope
FDA	Food and Drug Administration
FFB	final formulation buffer
GCP	Good Clinical Practices
GMT	geometric mean titer
gp	Glycoprotein
HAE	hereditary angioedema
HbsAg	hepatitis B surface antigen
HCV	hepatitis C virus
HIV	human immunodeficiency virus
HLA	human leukocyte antigen
IBC	Institutional Biosafety Committee
ICS	intracellular cytokine staining
IM	Intramuscular
IND	investigational new drug application
IRB	Institutional Review Board
LDL	low density lipoprotein
LIMS	Laboratory Information Management System
MCTU	Mobile Clinical Trials Unit
NIAID	National Institute of Allergy and Infectious Diseases

Abbreviation	Term
NIH	National Institutes of Health
NSAID	nonsteroidal anti-inflammatory drug
NVITAL	NIAID Vaccine Immune T-Cell and Antibody Laboratory
ORF	open reading frame
PBMC	peripheral blood mononuclear cells
PBS	phosphate buffered saline
PCR	polymerase chain reaction
pfu	plaque forming unit
Pol	Polymerase
PT	prothrombin time
PTT	partial thromboplastin time
PU	particle unit
QA	quality assurance
rAd5	recombinant adenoviral serotype 5 vector vaccine, VRC-HIVADV014-00-VP
RCA	replication-competent adenovirus
RSC	Regulatory Support Center
RPR	rapid plasma regain
SAE	serious adverse event
SD	standard deviation
SFU	spot-forming units
T1S1	transcriptionally inert spacer element
UNAIDS	Joint United Nations Programme on HIV/AIDS
VRC	Vaccine Research Center
WBC	white blood cell
WFI	water for injection

Table of IND and Protocol Numbers Discussed in VRC 015

IND Number	Vaccine(s)	VRC Protocol Identifier	NIH Protocol Number
BB-IND 11661	VRC-HIVADV014-00-VP	**VRC 006**<br>*HVTN 054*	04-I-0172
BB-IND 11894	VRC-HIVDNA009-00-VP prime<br>VRC-HIVADV014-00-VP boost	**VRC 009**<br>*HVTN 057*<br>*HVTN 068*<br>*HVTN 069*<br>*RV 156A*	05-I-0081
BB-IND12326	VRC-HIVDNA016-00-VP prime<br>VRC-HIVADV014-00-VP boost	**VRC 008**<br>**VRC 010**<br>**VRC 011**<br>*HVTN 204*<br>*IAVI-V001*<br>*RV-172*	05-I-0148<br>05-I-0140<br>06-I-0149
BB-IND 13358	VRC-HIVDNA044-00-VP<br>VRC-HIVADV038-00-VP<br>VRC-HIVADV027-00-VP	**VRC 012**<br>*HVTN 072*	07-I-0167

Précis

Protocol VRC 015: A Phase I, Open-Label Clinical Trial to Evaluate the Safety, Tolerability and Immunogenicity of a Multiclade Recombinant HIV-1 Adenoviral Vector Vaccine, VRC-HIVADV014-00-VP, in Uninfected Adults Randomized to Needle or Biojector Methods of Intramuscular Injection

Study Design: VRC 015 will examine safety, tolerability and immune response to the VRC recombinant adenoviral vector serotype 5 vector vaccine, VRC-HIVADV014-00-VP (rAd5), in uninfected subjects who will be randomized to receive the injection either by needle or Biojector injection. The study will include enrollment of rAd5 vaccine-naïve subjects, as well as enrollment of "rollover" subjects who received at least one rAd5 injection in a prior study that included the rAd5 vaccine. The hypothesis is that the rAd5 vaccine will be safe and immunogenic when administered by either needle or Biojector. The primary objectives are to evaluate the safety and tolerability of the rAd5 vaccine at 10^{10} PU dosage in the naïve and previously vaccinated, uninfected subjects when administered by needle or Biojectors. The secondary objectives include evaluating the HIV-1-specific humoral and T-cell immune responses and adenovirus serotype 5 (Ad5) antibody responses, and social impacts of participation in an HIV vaccine study. Exploratory evaluations include epitope mapping and other immunogenicity evaluations.

Product Description: The VRC HIV rAd5 vaccine is a recombinant product composed of 4 adenoviral vectors (in a 3:1:1:1 ratio) that encode the HIV-1 Gag/Pol polyprotein from clade B and HIV-1 Env glycoproteins from clades A, B, and C, respectively.

Subjects: Group 1 will include healthy adults, ages 18-50 years old who are HIV vaccine-naïve. Group 2 will include healthy adults, ages 18-55 years old, who are "rollover" enrollments from a prior study of the VRC HIV rAd5 vaccine.

Study Plan: Study Group 1 and Group 2 will be simultaneously enrolled. Both groups will be randomized in 1:1 ratio to receive the study injection by needle or Biojector. All study injections will be at a dosage of 10^{10} particle units (PU) of rAd5 vaccine delivered into deltoid muscle.

VRC 015	Subgroup	Number	Day 0 Injection
*Group 1 Vaccine-naïve	1a	10	10^{10} PU rAd5 IM needle
	1b	10	10^{10} PU rAd5 IM Biojector
**Group 2 Rollover enrollments	2a	5-10	10^{10} PU rAd5 IM needle
	2b	5-10	10^{10} PU rAd5 IM Biojector
	Total	30-40	(all deltoid muscle injections)

* Group 1 will include at least 8 with low (≤500 reciprocal titer) adenovirus serotype 5 antibody (Ad5Ab) and at least 8 with high (>500) Ad5Ab equally randomized into subgroups 1a and 1b; the remaining 4 subjects in Group 1 may be of any Ad5Ab titer.

** Group 2 rollover subjects are expected to be Ad5 seropositive from prior vaccine exposure; Ad5Ab titer will not be considered in the Group 2 randomization plan.

There are more than 100 past study participants who are potentially eligible for Group 2. The study design is based on at least 10 rollover subjects, but allows for additional rollover enrollment of up to 10 more (i.e., Group 2 total n=20) if there is greater than expected interest in participation. The sample for immunogenicity studies collected at Week 4 after vaccination from Group 2 subjects will be obtained by apheresis from those who are willing and eligible for apheresis; otherwise peripheral blood mononuclear cells (PBMCs) will be obtained from 80 mL blood collected by phlebotomy.

Study Duration: Subjects will be evaluated at 5 or more clinical visits for 24 weeks after the study injection and then followed by annual clinic, telephone or mail contact for the subsequent 4 years.

1. INTRODUCTION AND RATIONALE

1.1 HIV-1: Etiology, Disease Course, and Epidemiology

Globally, the human immunodeficiency virus (HIV) incidence rate is thought to have reached its peak in the late 1990s at over 3 million new cases per year. The reduction of HIV incidence is likely due to both prevention programs and the natural trend in an epidemic. The number of people living with HIV continues to grow due to population growth and longer life expectancy from improved access to antiretroviral medications. The Joint United Nations Programme on HIV/AIDS (UNAIDS) reports that in 2007 advances in the methodology resulted in a reduction in the estimated number of persons living with HIV/AIDS, but the difference in the estimates reported in 2006 and earlier are due to changes in methodology not trends in the epidemic itself. In the December 2007 UNAIDS report, it was estimated that 33.2 million [30.6 to 36.1] million people were living with HIV/AIDS globally. The 2007 global incidence was estimated to be 2.5 million [1.8-41. million] new cases per year. The estimated number of deaths due to AIDS in 2007 was 2.1 million [1.9-2.4 million] worldwide, of which 76% occurred in sub-Saharan Africa [1].

Beyond the human tragedy of HIV/AIDS, the costs of the epidemic pose a significant impediment to the economic growth and political stability of many countries. In developing countries and in segments of the U.S. population, anti-HIV therapies are frequently beyond financial reach. Accordingly, effective, low-cost tools for HIV prevention, such as vaccines, are urgently needed to bring the HIV epidemic under control. For this reason, the Vaccine Research Center (VRC) and Division of AIDS (DAIDS) at the National Institute of Allergy and Infectious Diseases (NIAID) of the National Institutes of Health (NIH) are committed to the development of safe, effective vaccines to prevent HIV infection and AIDS worldwide.

The use of multivalent vaccines, containing a defined mixture of immunogens from a number of prevalent subtypes should be a feasible approach to achieve broadly-protective HIV vaccines. The World Health Organization UNAIDS HIV Vaccine Advisory Committee has recommended that candidate HIV vaccines be designed based upon the strains prevalent in the country in which trials are to be conducted [2]. The Vaccine Research Center, NIAID, NIH and the World Health Organization-Joint United Nations Programme on HIV/AIDS organized a meeting focused on the genetic diversity of HIV and strategies to develop vaccine candidates. A consensus was reached that established multiclade candidate vaccines as a high international scientific priority [3]. The recombinant adenoviral serotype 5 vector vaccine, VRC-HIVADV014-00-VP, encodes *gag* and *pol* gene sequences from clade B as well as *env* genes from clades A, B and C. Clades A, B and C together represent the viral subtypes responsible for about 75%-85% of new HIV infections in the world [4, 5].

1.2 Previous Experience with the Study Vaccine, VRC-HIVADV014-00-VP

The Vaccine Research Center (VRC), NIAID, NIH, in collaboration with DAIDS, NIAID, NIH has conducted multiple trials with the recombinant adenoviral serotype 5 vector vaccine, VRC-HIVADV014-00-VP (rAd5), to be used in this study. In this protocol the abbreviation rAd5 will refer specifically to the VRC candidate vaccine, VRC-HIVADV014-00-VP. Results of the VRC 006 study, a 36-subject, Phase I, double-blind, placebo-controlled, dose escalation study of the rAd5 vaccine, have been published [6]. As of January 2008 more than 1300 subjects (see **Table 1.1**) have been enrolled in Phase I or Phase II clinical trials involving this product; of these at

least 800 subjects have received the rAd5 vaccine alone or as a booster injection and the majority of vaccinations have been administered at the 10^{10} PU dose. When the rAd5 vaccine has been administered as a booster injection, the priming vaccinations have usually been with a multiclade DNA vaccine. However, two studies (HVTN 068 and VRC 011) were designed to include a group of 30 participants each of whom received an rAd5 prime with an rAd5 boost. The studies are shown in Table 1.1 and all are completed except for long-term contacts that are ongoing in some studies. Across all studies to date, the rAd5 vaccine has been well tolerated and immunogenic whether administered alone or as a booster injection in studies to date. The adverse events generally associated with administration of the rAd5 vaccine are described in Section 8.2.1 of the protocol.

Table 1.1 Clinical Trials with the VRC rAd5 Vaccine for Uninfected Participants

IND	Product	Protocol Number	Protocol Status	Total Accrual
BB11661	VRC-HIVADV014-00-VP	VRC 006	Completed; long-term contacts ongoing	*36
		HVTN 054	Completed	*48
BB11894	VRC-HIVDNA009-00-VP VRC-HIVADV014-00-VP	HVTN 057	Completed	*70
		VRC 009	Completed	10
		HVTN 068	Completed; long-term contacts ongoing	*66
		HVTN 069	Completed; long-term contacts ongoing	90
		RV156A	Completed; long-term contacts planned	18
BB12326	VRC-HIVDNA016-00-VP VRC-HIVADV014-00-VP	VRC 008	Completed	40
		VRC 010	Completed	4
		VRC 011	Completed; long-term contacts ongoing	60
		IAVI V001	Completed; long-term contacts ongoing	*114
		RV 172	Completed; long-term contacts ongoing	*326
		HVTN 204	Completed; long-term contacts ongoing	*480
Total Accrual<br>*Studies with asterisk include some placebo recipients<br>Completed = all required clinic visits are completed and clinical database locked				1362

1.3 PREVIOUS EXPERIENCE WITH OTHER RAD5 HIV VACCINES

An adenovirus serotype 5 vector vaccine developed as a preventive HIV vaccine by Merck had been in clinical trials for several years until September 2007 when further evaluation of this vaccine was halted. The VRC-rAd5 HIV vaccine is different in several ways from the Merck (MRK-Ad5) HIV vaccine, including the adenovirus genes deleted from the vector, the cell line in which the vaccine is produced, the encoded HIV antigens, the immunogenicity characteristics in humans and the efficacy profile in nonhuman primate studies. Nonetheless, the interim results of the Phase IIB efficacy study of the Merck-Ad5 vaccine (HVTN 502, also known as the Step Study) have been considered in the plan for this protocol. The Step Study results suggest caution in administering Ad5 vector vaccines to subjects with pre-existing Ad5 antibody (Ab) at enrollment [7]. The Step Study was designed to enroll 3000 men and women with an increased risk of exposure to HIV infection. The Step Study was halted on September 19, 2007 for futility when the interim data reviewed by the Data and Safety Monitoring Board (DSMB) indicated that

the MRK-Ad5 HIV vaccine was not preventing HIV infections and was not reducing the HIV viral load in participants who became HIV-infected. An unexpected safety concern was that there were more HIV infections in male vaccinated participants who already had Ad5 Ab at the time of enrollment (from a prior Ad5 naturally occurring infection) than the male placebo recipients from the same group. There was only one HIV infection among the women in the Step Study, therefore this trend was observed only in men. Although the MRK-Ad5 vaccine induces Ad5-specific Ab in participants who are Ad5-seronegative at enrollment, and three doses were given, this group did not have different rates of HIV infection between the vaccine and placebo groups. Further evaluations of Step Study results are ongoing to try to identify the basis for this unexpected observation.

In October 2007, NIAID and the HIV Vaccine Trials Network (HVTN) also stopped immunizations and enrollment in the HVTN 503 ("Phambili") study in South Africa of the MRK-Ad5 HIV vaccine. At the AIDS Vaccine 2008 Conference (Cape Town, South Africa) data as of September 28, 2008 were presented as follows: among the 801 participants (360 women and 441 men), there were 29 confirmed HIV infections (22 women and 7 men). Of these 17 were in the vaccine group (16 Ad5 seropositive and 1 Ad5 seronegative) and 12 in the placebo group (9 Ad5 seropositive and 3 Ad5 seronegative). Due to the limited sample size and unblinding that occurred at the time the study was halted it may not be possible to draw any scientifically valid conclusions from this study about the vaccine's effect on HIV acquisition.

In the Step Study of the Merck-Ad5 HIV vaccine three factors co-existed in the group in which a trend of increased HIV acquisition was observed: 1) pre-existing immunity to adenovirus serotype 5 from natural infection; 2) high risk for HIV exposure and 3) receipt of the MRK-Ad5 vaccine. There was not evidence of increased HIV acquisition in the absence of any one of these factors. It is also unknown if this trend will remain over the long-term follow-up of this group and also whether the trend is due to the prior Ad5 immunity or is due to another factor for which prior Ad5 infection may be a demographic marker rather than the cause. The VRC views the potential increased risk of HIV acquisition following HIV exposure that was raised by the Step Study can be addressed in the VRC 015 protocol by enrolling only subjects at low risk for HIV exposure, carefully counseling subjects about the need to not engage in activities that risk HIV exposure, and continuing risk reduction counseling throughout the study. In addition, subjects will be tested for HIV to document HIV serostatus.

1.4 RATIONALE FOR EVALUATING BIOJECTOR ADMINISTRATION OF RAD5 VACCINE

To date, all of the VRC rAd5 vaccinations in prior clinical trials have been administered by needle and syringe. This study will be the first Phase I evaluation of administration of this vaccine by the Biojector® 2000 Needle-Free Injection Management System (Biojector). The most direct comparison between needle and Biojector administration will be accomplished with the subjects in Group 1 who will all be HIV vaccine naïve and randomized at enrollment to method of injection. Group 2 subjects will also be randomized at enrollment to a method of injection and provide additional data to help in the assessment of needle as compared to Biojector rAd5 injections, however, due to the variety of prior HIV vaccine types (i.e., rAd5 alone, 4-plasmid DNA prime-rAd5 boost, 6-plasmid DNA prime-rAd5 boost, or rAd5 prime-rAd5 boost), prior rAd5 dosage (10^9, 10^{10}, or 10^{11}), and routes of administration (IM, SC, ID) and the at various intervals of time in the past that these subjects will have experienced their prior vaccine injections, these data will be more exploratory in nature.

Use of the Biojector for administration of other vaccines has demonstrated effective immune responses, and although its use is associated with some self-limited pain, mild bruising, a small laceration and redness or swelling at the injection site, this method of administration is well tolerated, and offers the advantage of eliminating needlestick accidents in the clinic [8]. This system has U.S. Food and Drug Administration (FDA) clearance for delivering intramuscular (IM) injections of vaccine. When the Biojector actuator is depressed, CO_2 is released from a replaceable, multi-dose cartridge, causing the plunger to push the study product out of the sterile single-use syringe through the skin and into the underlying tissue. The study product is expelled through a micro-orifice at high velocity in a fraction of a second to pierce the skin and is designed to prevent tissue splashback.

The VRC has extensive experience with use of the Biojector for administration of plasmid DNA vaccines. The VRC 008 study provided the opportunity to evaluate whether or not DNA vaccine administered by Biojector had any effect on magnitude of immune response compared to needle injection of the same vaccine. The VRC 008 schedule included three VRC-HIVDNA016-00-VP (DNA vaccine) injections at Weeks 0, 4 and 8, followed by one rAd5 booster (all by needle injection) at Week 24. Forty subjects, half with high (>1:500) and half with low (≤1:500) adenovirus serotype 5 antibody titers, were randomized in a 1:1 ratio to receive the DNA vaccinations by either needle and syringe (N/S) or by Biojector and were also randomized in a 1:1 ratio to receive the booster vaccination with either 10^{10} PU or 10^{11} PU of the rAd5 vaccine. All 120 of the planned DNA prime vaccinations and 39 of the rAd5 booster injections were administered. **Figure 1** shows a side-by-side comparison of the median magnitude of ELISpot responses for the Biojector and N/S primed groups, at baseline (Day 0), after two DNA injections (Weeks 6 and 8), post prime (Weeks 10, 12 and 24) and post rAd5 boost (Weeks 28, 30 and 42). The stacked bars represent the summed medians of magnitude of response to Env, Gag, Pol and Nef peptide pools (from bottom to top). Arrows indicate the study injection timepoints for DNA vaccine at Weeks 0, 4, 8 and rAd5 vaccine at Week 24. The frequency of IFN-γ ELISpot responses for any peptide pool at 4 weeks post rAd5 boost were 17/19 (89%) for the Biojector primed group and 13/17 (76%) for the N/S primed group. At Week 28, the cumulative median ELISpot response (sum of Gag+Pol+Nef+highest Env responses) was higher (p=0.02) in the Biojector primed group than in the N/S primed group.

Figure 1. VRC 008 ELISpot Responses in Subjects Receiving DNA Vaccine by Biojector as Compared to by Needle Injection.

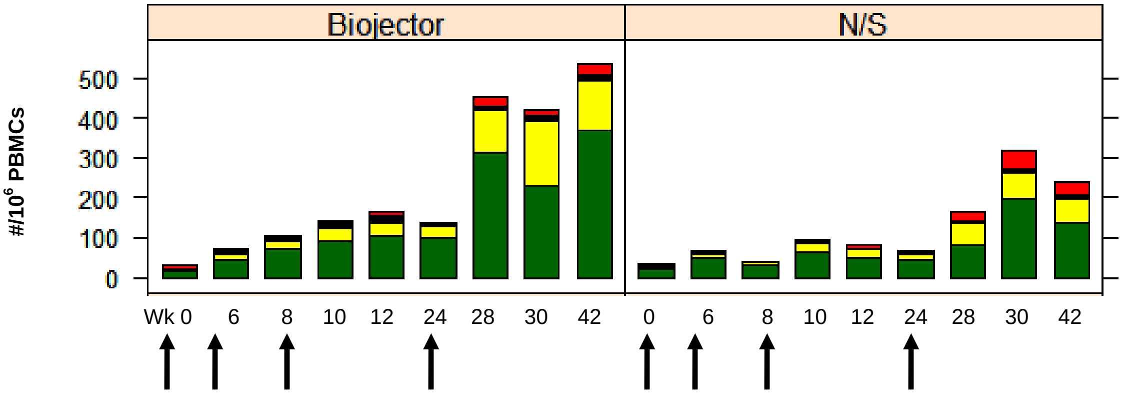

Given the VRC 008 results suggesting that magnitude of response is affected by the device used for the DNA prime injections, it will be worthwhile to obtain preliminary evidence of whether or not the magnitude of immune response to the rAd5 injection can also be increased, on average, by use of Biojector.

Previous Phase I clinical trials for evaluation of safety and immunogenicity of live viral vectors administered by Biojector include investigational vaccinia and canarypox vectors expressing human carcinoembryonic antigen (CEA) in patients with advanced cancer. Replication-deficient ALVAC-CEA canarypox vector was administered intramuscularly by Biojector into the buttock, arm, or thigh of 20 patients with Stage IV adenocarcinoma at doses ranging from 2.5 x 10^5 to 2.5 x 10^7 plaque forming units (pfu) and found to increase the frequency of CEA-specific cytotoxic T-lymphocytes (CTL) 3.7-fold [9], although no antitumor effects were observed and no dose response was observed. Mild injection site soreness was occasionally reported. These investigators reported better increases in immune response following an intradermal vaccinia prime followed by a subcutaneous canarypox boost (delivered by Biojector)–CEA boost vaccination regimen in 18 patients with advanced cancer [10]. This regimen was also well tolerated.

Preclinical evaluations have provided data comparing Biojector and needle administration of viral vectors. In a study in hairless guinea pigs, Biojector injections of adenovector serotype 2-expressing beta-galactosidase showed comparable gene expression and cellular immune responses to intradermal needle administration. Biojector injections were evaluated as significantly enhancing the humoral immune responses (GenZyme Molecular Oncology and Biojector, *personal communications*). This study suggested that the viral vector retained integrity and remained immunogenic when delivered by Biojector.

Studies have been conducted in mice evaluating Biojector delivery of modified vaccinia virus Ankara expressing herpes simplex virus 2 transmembrane deleted glycoprotein D as part of DNA-MVA vector injection regimens. The Biojector delivery of the MVA vector intradermally elicited similar specific antibody response to subcutaneous needle delivery and Biojector delivery was assessed as efficient and reproducible in eliciting a strong immune response [11].

Although not published in a peer-reviewed journal, information about injection of a rAd5 vaccine encoding Gag developed by Merck is available on a World Intellectual Property Organization website. The summary at this website includes the information that an experiment in rhesus monkeys compared a dose titration of vaccine, as well as needle vs. Biojector delivery at most doses. Anti-gag ELISpot responses were measured in all monkeys at eight weeks. Nearly all vaccinated monkeys developed significant gamma-interferon responses to this vaccine, although prior exposure to adenovirus reduced response levels, and a dose response appears to have been obtained with the highest doses giving the best responses. In this experiment (as well as in an independent experiment) no difference was observed for needle vs. Biojector delivery of vaccine. $CD4^+$ T cell depletion of these samples showed that the ELISpot responses are largely due to $CD8^+$ T cells [12].

In VRC 015 the two Groups (rAd5 vaccine-naïve subjects and subjects receiving booster injections) will be separately evaluated for immune responses. The magnitude of immune response may be affected by prior exposure to vaccine-delivered HIV antigens. **Table 1.2** shows the frequency and magnitude of cellular immune response, as assessed by enzyme-linked immunospot (ELISpot) assay at 4 weeks after a single 10^{10} PU rAd5 vaccination in VRC 006 and

4 weeks after a single 10^{10} rAd5 booster injection in VRC 009. The greatest frequency and magnitude of response was detected by stimulation with peptide pools representing the EnvA antigen.

Table 1.2 Frequency and Magnitude of Positive T Cell Responses after Vaccination as Assessed by ELISpot for rAd5 alone (VRC 006) and rAd5 as booster (VRC 009) Administered by Needle and Syringe at 10^{10} PU

ELISpot	VRC 006 (10^{10} PU dose) Single agent		VRC 009 (10^{10} PU dose) booster	
Peptide Pool	Frequency [95% CI]	Mean SFU/10^6 PBMC (log_{10} GMT) [95%CI] *n*=10	Frequency [95% CI]	Mean SFU/10^6 PBMC (log_{10} GMT) [95%CI] *n*=10
Env (A)	6/10 = 60% [26%, 88%]	112.17 (1.79) [1.45, 2.12]	10/10 = 100% [69%, 100%]	1337.67 (2.82) [2.41, 3.24]
Env (B)	4/10 = 40% [12%, 74%]	79.67 (1.75) [1.46, 2.03]	10/10 = 100% [69%, 100%]	1306.17 (2.76) [2.31, 3.20]
Env (C)	3/10 = 30% [7% , 65%]	57.50 (1.60) [1.32, 1.88]	9/10 = 90% [55%, 100%]	376.83 (2.35) [2.00, 2.69]
Gag (B)	2/10 = 20% [3% , 56%]	23.33 (1.10) [0.65, 1.55]	7/10 = 70% [35%, 93%]	188.33 (1.95) [1.56, 2.35]
Pol (B)-1	2/10 = 20% [3% , 56%]	124.67 (1.13) [0.51, 1.76]	3/10 = 50% [7%, 65%]	86.67 (1.44) [0.99, 1.89]
Pol (B)-2	2/10 = 20% [3% , 56%]	27.50 (1.30) [0.99, 1.60)	2/10 = 20% [3%, 56%]	28.67 (1.26) [0.92, 1.59]
Any	7/10 = 70% [35%, 93%]		10/10 = 100% [69%, 100%]	

1.5 MEASURES OF IMMUNOGENICITY

In this Phase I study, the Week 4 samples collected from Group 1 subjects (i.e., those who are HIV-vaccine naïve) will be the primary timepoint at which the immunogenicity of VRC-HIVADV014-00-VP when administered by needle or by Biojector will be evaluated by employing ELISpot and intracellular cytokine staining (ICS) assays, as well as assays that evaluate HIV-specific antibody responses. Clade-specific peptides will be used to detect T-cell responses by an ELISpot assay based on a previously published method [13]. The ICS assay is based upon previously published methods [14] and quantitates the frequency of $CD4^+$ and $CD8^+$ cells that produce interleukin-2 (IL-2) and/or interferon-gamma (IFN-γ) in response to pools of overlapping peptides representing HIV antigens (Gag, Pol, Nef or Env) from specific HIV clades.

The pre-existing and post-vaccination presence of adenovirus serotype 5 neutralizing antibody in study volunteers will be evaluated using a previously published luciferase transgene detection method [15]. Other assays may also be completed from stored samples at a later date if further elucidation of immunogenicity is of interest.

Whenever possible, the Week 4 samples from Group 2 subjects (i.e., those who are receiving a booster HIV vaccine injection) will be obtained by apheresis. When apheresis is not possible in this timeframe, 80 mL of blood will be collected by phlebotomy in order to complete the immunogenicity assessments. About 4 weeks after vaccination is anticipated to be the peak of T cell response. The larger number of PBMCs that can be obtained by apheresis will permit additional epitope mapping of immune responses to elucidate the breadth of the vaccine-induced T cell response post-boosting. A two-pass apheresis procedure yields about 1×10^9 (1 billion) PBMC with only a 20 mL volume blood loss from the procedure, whereas an 80 mL phlebotomy yields only about 8×10^7 (80 million) PBMCs. Although all subjects will provide valuable information about the immunological response to the adenoviral vector booster vaccination, more extensive immunological analysis of a booster vaccination will be possible for those subjects who are willing and able to undergo apheresis about 4 weeks after the study vaccination.

2. BACKGROUND ON VACCINE

2.1 ADENOVIRAL VECTORS IN VRC-HIVADV014-00-VP

VRC-HIVADV014-00-VP (rAd5) is a replication-deficient, combination vaccine containing four recombinant adenoviral vectors. These vectors contain gene sequences that code for clade B HIV-1 Gag and Pol as well as clade A, clade B, and clade C Env proteins. *In vitro* expression by these vectors produces immunogens that induce an immune response against HIV. The envelope genes were chosen as representative primary isolates from each of the three clades.

The process for constructing the four recombinant adenoviral vectors is based upon a rapid vector construction system (AdFAST™, GenVec, Inc.) used to generate adenoviral vectors that express the four HIV antigens gp140(A), gp140(B)dv12, gp140(C) and GagPol(B) driven by the cytomegalovirus (CMV) immediate-early promoter [16]. Manufacturing is based upon production in a proprietary cell line (293-ORF6), yielding adenoviral vectors that are replication deficient. The product is formulated as a sterile liquid injectable dosage form for intramuscular injection.

The GV11 adenoviral backbone was chosen to reduce the risk of replication-competent adenovirus (RCA) generation during clinical production. The GV11 backbone contains deletions of two essential regions, E1 and E4, as well as a partial E3 deletion that render the vaccine product replication-deficient. The generation of RCA would require two independent recombination events in a single adenovirus genome, predicted to be an extremely rare event [17].

The Ad_{GV} (HIV).11D vectors contain HIV-1 antigen open reading frame (ORF) expression cassettes inserted to replace the deleted adenovirus E1 gene region. Other deleted adenovirus regions include a partial E3 and all of E4, which has been replaced with a transcriptionally inert spacer element (T1S1) that enhances production of the adenoviral vectors [18].

The 293-ORF6 cell line used to propagate these E1, E4 and partial E3 deleted vectors was developed at GenVec, Inc. These cells were constructed by stably transforming 293 cells (which are of human embryonic kidney origin) with an inducible E4-ORF6 expression cassette [19]. This enables the cells to efficiently complement the E1-, E4-, and partial E3-deleted adenoviral vectors, provide increased transgene capacity and greatly reduce the potential to generate replication-competent adenovirus. An assay for replication-competent adenovirus is performed

in the final release testing for all vectors; RCA has not been observed in this packaging system during the manufacture of multiple gene-based products.

Purified adenoviral vector serves as a vector bank for subsequent production of the four vaccine adenoviral vectors. This vector bank is tested for sterility, mycoplasma and other adventitious agents prior to its being used for manufacturing of clinical supplies. The four vaccine adenoviral vectors are generated by introducing a DNA plasmid consisting of the adenoviral genome into the 293-ORF6 cells. The adenoviral vector in the lysate from the transfected cells is serially passaged to expand the titer of adenoviral vector. The identity and integrity of the passages is verified by polymerase chain reaction (PCR) and expression of the HIV-1 gene is confirmed by Western Blot analysis. Purified adenoviral vector is produced by infecting the 293-ORF6 cells with the adenoviral vector in the lysate; after the infection of the cells is complete, the material is collected and the vector is purified from the cells.

To date, the lots of the rAd5 vaccine administered in clinical trials have been manufactured by a process using a serum-free suspension shaker flask culture system, and the four vaccine adenoviral vectors were purified using a cesium chloride (CsCl) gradient centrifugation process. CsCl was removed by dialyzing the virus preparation against the final formulation buffer (VRC-DILUENT013-DIL-VP). The lot of rAd5 vaccine that will be administered in this study was manufactured using a serum-free suspension bioreactor culture system and column chromatography purification. The manufacturing process has been modified primarily to scale up the production process. To accomplish this, changes were made in the concentration and purification stages of vaccine manufacturing. The product has undergone potency testing in animals, expression testing *in vitro* and standard lot release testing.

2.2 MANUFACTURE OF THE FINAL VACCINE PRODUCT

The candidate vaccine is produced under current Good Manufacturing Practices (cGMP). The multiclade adenoviral vector vaccine product, VRC-HIVADV014-00-VP, is a 3:1:1:1 ratio of the adenoviral vectors that encode for HIV-1 Gag/Pol polyprotein from clade B and HIV-1 Env glycoproteins from clades A, B, and C, respectively. DNA plasmids developed by the VRC/NIAID/NIH (Bethesda, MD) were used to construct the adenoviral vector clinical seed stocks used to produce the vaccine. The construction of the adenoviral vectors has been published [16, 19] and is described in the Investigators' Brochure. The dosage is specified in particle units (PU). PUs are the number of viral particles, active or not, found in the product as determined by spectroscopy. The adenoviral vectors were manufactured by GenVec, Inc. (Gaithersburg, MD) at a contract manufacturer, Molecular Medicine, San Diego, CA. Final formulation, fill and packaging were performed by the VRC/NIAID Vaccine Pilot Plant.

Specific manufacturing information is included on the product vial labels and Certificates of Analysis. QA lot release testing by the manufacturer verifies conformance to product specifications prior to use in clinical trials.

The final formulation buffer (FFB) is composed of sodium chloride, Tris buffer, trehalose•$2H_2O$ (low endotoxin), magnesium chloride•$6H_2O$, monooleate (Tween 80) and water for injection (WFI). FFB was custom manufactured by Cambrex (Walkerville, MD).

3. STUDY OBJECTIVES

3.1 PRIMARY OBJECTIVES

- To evaluate the safety and tolerability of the VRC candidate HIV rAd5 vaccine at 10^{10} PU administered IM by Biojector or by needle injection in uninfected vaccine-naïve subjects.
- To evaluate the safety and tolerability of the VRC candidate HIV rAd5 vaccine at 10^{10} PU administered IM by Biojector or by needle injection in uninfected subjects who previously received at least one injection of this vaccine at any dosage or route of administration.

3.2 SECONDARY OBJECTIVES

- To evaluate the immunogenicity of rAd5 at a dosage of 10^{10} PU IM as measured by T cell responses to vaccine-derived peptide pools (ELISpot or ICS) at 4 weeks after administration by needle or by Biojector in vaccine-naïve subjects.
- To evaluate the immunogenicity of rAd5 as a booster injection at a dosage of 10^{10} PU IM as measured by T cell responses to vaccine-derived peptide pools (ELISpot or ICS) at 4 weeks after administration by needle or by Biojector in subjects previously vaccinated with rAd5 at any dosage.
- To evaluate adenovirus serotype 5 neutralizing antibody titers at baseline and 4 weeks after the rAd5 injections are administered.
- To monitor the social impact of participating in an HIV-1 vaccine study.

3.3 EXPLORATORY OBJECTIVES

- To describe the frequency and magnitude of ICS, ELISpot, vaccine antigen-specific ELISA and other immunological responses to the rAd5 vaccine over time in the naïve and rAd5 boosted subjects.
- To compare the magnitude of immune responses to the rAd5 vaccine when administered by Biojector and by needle injection.
- To evaluate adenovirus serotype 5 neutralizing antibody titers throughout the study.
- To perform epitope mapping of the $CD8^+$ and $CD4^+$ T cell responses using ELISpot and multicolor flow cytometric analysis at Week 4 after the study injection in rAd5 boosted subjects.
- To obtain PBMC from vaccine recipients with good immunologic responses to assist in the validation of research assays.

4. STUDY DESIGN

This study will examine safety, tolerability and immune response to the VRC recombinant adenoviral serotype 5 (rAd5) vector vaccine in uninfected subjects who will be randomized to receive the injection either by needle or Biojector injection. The study will include enrollment of two Groups simultaneously. Group 1 will enroll rAd5 vaccine-naïve subjects and Group 2 will enroll "rollover" subjects who received at least one rAd5 injection (at any dosage or by any route of administration) in a prior rAd5 vaccine study; the prior study regimen may also have included an HIV-1 DNA vaccine. The schema is shown in Table 4.1.

Table 4.1 VRC 015 Study Injection Schedule

VRC 015	Subgroup	Number	Day 0 Injection
*Group 1 Vaccine-naive	1a	10	10^{10} PU rAd5 IM needle
	1b	10	10^{10} PU rAd5 IM Biojector
**Group 2 Rollover enrollments	2a	5-10	10^{10} PU rAd5 IM needle
	2b	5-10	10^{10} PU rAd5 IM Biojector
Total		30-40	(all deltoid muscle injections)

* Group 1 will include at least 8 with low (≤500 reciprocal titer) adenovirus serotype 5 neutralizing antibody (Ad5Ab) and at least 8 with high (>500) Ad5Ab equally randomized into subgroups 1a and 1b; the remaining 4 subjects in Group 1 may be of any Ad5Ab titer.

** Group 2 rollover subjects are expected to be Ad5 seropositive from prior vaccine exposure; Ad5Ab titer will not be considered in the Group 2 randomization plan. Additional rollover enrollments of up to total n=20 is permitted if there is greater than expected interest in study participation.

Assignment to needle or Biojector injections will not be known to staff or subjects prior to enrollment, but will become known to both study staff and subjects at the time enrollment is completed.

The hypothesis is that the rAd5 vaccine will be safe and immunogenic when administered by either needle or Biojector.

The Group 1 consideration of Ad5Ab titer in the randomization plan is intended to ensure some balance in the needle and Biojector subgroups when comparing immune responses.

Safety of the vaccine regimens will be evaluated at scheduled study visits and by study subject report. Specimens to evaluate immunogenicity will be taken at baseline and at specified timepoints. The HIV-1-specific immune responses will be assessed by cellular immune function assays and humoral immunity assays. The study subjects will require 24 weeks on study to complete the vaccination and follow-up. After Week 24, subjects will have an annual follow-up by clinic visit, telephone or mail for an additional 4 years.

4.1 STUDY POPULATION

All study activities will be carried out by the VRC Clinic at the NIH Clinical Center or its approved satellite facilities [e.g., Mobile Clinical Trials Unit (MCTU) or VRC Clinic at Cedar Lane]. Vaccinations will only occur at the NIH Clinical Center. Healthy, HIV-uninfected volunteers will be recruited through Institutional Review Board (IRB)-approved advertising and will be screened through VRC 000 (02-I-0127), a screening protocol for healthy volunteers who are interested in participating in HIV vaccine clinical trials, to confirm eligibility requirements for participation. The screening and education process required prior to enrollment should ensure that subjects comprehend the purpose and details of the study. Some eligibility requirements have specified parameters that differ for Group 1 and Group 2.

Prior to signing the VRC 015 informed consent, eligible volunteers will take a short "Assessment of Understanding" quiz to test understanding of this vaccine study. Incorrect answers will be explained to the volunteer and they will sign the informed consent document only after the Study Coordinator is satisfied with their understanding of the study.

4.1.1 Inclusion Criteria

A participant must meet all of the following criteria:

1. 18 to 50 years old if enrolling into Group 1 or 18 to 55 years old if enrolling into Group 2.
2. HIV vaccine naïve if enrolling into Group 1, or receipt of the VRC HIV rAd5 vaccine in a previous study without experiencing a serious adverse event attributed (i.e., definitely, probably, possibly, or probably not related) to the vaccine if enrolling into Group 2.
3. Available for clinical follow-up through Week 24 of the study and committed to four years of annual follow-up contact after Week 24.
4. Able to provide proof of identity to the satisfaction of the study clinician completing the enrollment process.
5. Complete an Assessment of Understanding that includes understanding of the STEP Study results prior to enrollment and verbalize understanding of all questions answered incorrectly.
6. Able and willing to complete the informed consent process.
7. Willing to receive HIV test results and willing to abide by NIH guidelines for partner notification of positive HIV results.
8. Willing to donate blood for sample storage to be used for future research.
9. Willing to discuss HIV infection risks, amenable to risk reduction counseling, committed to maintaining behavior consistent with low risk of HIV exposure through the last required protocol clinic visit, and assessed by the clinic staff as being at low risk of HIV infection on the basis of behaviors in the 12 months before enrollment as follows:

Sexually abstinent<br><br>**OR**<br><br>Had two or fewer mutually monogamous relationships with partners believed to be HIV-uninfected and who did not use injection drugs, crack cocaine or methamphetamine<br><br>**OR**<br><br>Had two or fewer partners believed to be HIV-uninfected and who did not use injection drugs, crack cocaine or methamphetamine and with whom he/she regularly used condoms for vaginal or anal intercourse

10. In good general health without clinically significant medical history.

11. Physical examination and laboratory results without clinically significant findings and a body mass index (BMI) ≤ 40 within the 56 days prior to enrollment.

Laboratory Criteria within 56 days prior to enrollment:

12. Hemoglobin ≥ 11.5 g/dL for women; ≥13.0 g/dL for men.

13. White blood cells (WBC) = 3,300-12,000 cells/mm^3.

14. Differential either within institutional normal range or accompanied by site physician approval.

15. Total lymphocyte count ≥ 800 cells/mm^3.

16. Alanine aminotransferase (ALT) ≤ 1.25 x upper limit of normal.

17. Serum creatinine ≤ 1 x upper limit of normal (≤1.3 mg/dL for females; ≤1.4 mg/dL for males).

18. HIV-uninfected as evidenced by a negative FDA-approved HIV diagnostic blood test if enrolling into Group 1 or negative HIV polymerase chain reaction (PCR) test if enrolling into Group 2

19. Negative hepatitis B surface antigen (HbsAg).

20. Negative anti-HCV (hepatitis C virus antibody) and negative HCV PCR.

Female-Specific Criteria:

21. Negative β-HCG (human chorionic gonadotropin) pregnancy test (urine or serum) on day of enrollment for women presumed to be of reproductive potential.

22. A female participant must meet any of the following criteria:

    No reproductive potential because of menopause [one year without menses] or because of a hysterectomy, bilateral oophorectomy, or tubal ligation,

    or

    Participant agrees to be heterosexually inactive at least 21 days prior to enrollment and through Week 24 of the study,

    or

    Participant agrees to consistently practice contraception at least 21 days prior to enrollment and through Week 24 of the study by one of the following methods:

    - condoms, male or female, with or without a spermicide
    - diaphragm or cervical cap with spermicide
    - intrauterine device

- contraceptive pills or patch, Depo-Provera or other FDA-approved contraceptive method
- male partner has previously undergone a vasectomy.

4.1.2 Exclusion Criteria

A volunteer will be excluded if one or more of the following conditions apply:

Women:

1. Woman who is breast-feeding or planning to become pregnant during the 24 weeks of study participation.

Volunteer has received any of the following substances:

2. Immunosuppressive medications, cytotoxic medications, inhaled corticosteroids, or long-acting beta-agonists within the past three months.
   [Note: The following will NOT exclude study participation:
   - use of corticosteroid nasal spray for rhinitis;
   - topical corticosteroids for an acute uncomplicated dermatitis;
   - short-acting beta-agonists in controlled asthmatics; a short course (10 days or less) of corticosteroids for a non-chronic condition completed at least 2 weeks prior to enrollment in this study]

3. Blood products within 112 days (16 weeks) days prior to HIV screening.

4. Immunoglobulin within 56 days (8 weeks) prior to HIV screening.

5. Investigational research agents within 28 days (4 weeks) prior to initial study vaccine administration.

6. Live attenuated vaccines within 28 days (4 weeks) prior to initial study vaccine administration.

7. Medically indicated subunit or killed vaccines, e.g. influenza, pneumococcal, or allergy treatment with antigen injections, within 14 days (2 weeks) prior to study vaccine administration.

8. Current anti-tuberculosis prophylaxis or therapy.

Volunteer has a history of any of the following clinically significant conditions:

9. Serious adverse reactions to vaccines such as anaphylaxis, urticaria (hives), respiratory difficulty, angioedema, or abdominal pain.

10. Autoimmune disease or immunodeficiency.

11. Asthma that is unstable or required emergent care, urgent care, hospitalization or intubation

during the past two years or that requires the use of oral or intravenous corticosteroids.

12. Diabetes mellitus (type I or II), with the exception of gestational diabetes.

13. Thyroid disease that is not well controlled.

14. A history of hereditary angioedema (HAE), acquired angioedema (AAE), or idiopathic forms of angioedema.

15. Generalized idiopathic urticaria within the last 1 year.

16. Hypertension that is not well controlled by medication or is more than 145/95 at enrollment.

17. Bleeding disorder diagnosed by a doctor (e.g. factor deficiency, coagulopathy, or platelet disorder requiring special precautions) or significant bruising or bleeding difficulties with IM injections or blood draws.

18. Within the 12 months prior to enrollment: newly-acquired syphilis, gonorrhea, non-gonococcal urethritis, herpes simplex virus type 2, chlamydia, pelvic inflammatory disease, trichomonas, mucopurulent cervicitis, epididymitis, proctitis, lymphogranuloma venereum, chancroid, or hepatitis B.

19. Malignancy that is active or treated malignancy for which there is not *reasonable* assurance of sustained cure or malignancy that is likely to recur during the period of the study.

20. Seizure disorder other than: 1) febrile seizures, 2) seizures secondary to alcohol withdrawal more than 3 years ago, or 3) seizures that have not required treatment within the last 3 years.

21. Asplenia, functional asplenia or any condition resulting in the absence or removal of the spleen.

22. Psychiatric condition that precludes compliance with the protocol; past or present psychoses; past or present bipolar disorder; disorder requiring lithium; or within five years prior to enrollment, history of a suicide plan or attempt.

23. Any medical, psychiatric, social condition, occupational reason or other responsibility that, in the judgment of the investigator, is a contraindication to protocol participation or impairs a volunteer's ability to give informed consent.

24. Within the 12 months prior to enrollment, one or more of the following:
    - excessive daily alcohol use
    - frequent binge drinking
    - chronic marijuana abuse
    - any other illicit drug use

4.2 CLINICAL PROCEDURES AND LABORATORY ASSAYS

Evaluation of the safety of this vaccine will include laboratory studies, medical history, physical assessment by clinicians, and subject self-assessment entered on a diary card for 5 days following the injection. Assays for immune responses will be performed at the Vaccine Research Center. The clinical procedures are described in Section 4.2.2 and the schedule is provided in Appendix III. Total blood volume drawn from each subject will not exceed the NIH Clinical Center Guidelines.

4.2.1 Screening

Screening for this study will be completed through the Vaccine Research Center's Screening Protocol, VRC 000 (NIH 02-I-0127). The evaluations and sample collection that will be included in VRC 000 screening are a medical history, physical exam, any laboratory tests needed to confirm eligibility, pregnancy test (for females of reproductive potential), and questions regarding sexual behavior and other practices. The adenovirus serology used in the randomization for Group 1 subjects must be obtained within 12 weeks (84 days) prior to enrollment. Any of the tests done during screening that are needed for a specific eligibility requirement must be done within the window needed to meet study eligibility. Risk status for HIV infection will be determined by a series of questions designed to identify risk factors. Storage samples of PBMCs, plasma and serum will also be collected. General eligibility for clinical trials will be dependent on results of laboratory tests and answers to the interview questions. Informed consent documents for vaccine trials will be reviewed, and counseling relating to the potential risks of becoming pregnant during this trial and avoiding HIV infection will be provided. An Assessment of Understanding of VRC 015 is completed on the day the subject is scheduled to enroll in VRC 015.

4.2.2 Day 0 through Week 24

Day 0 is defined as the day of VRC 015 enrollment and study injection. VRC 015-specific eligibility is reviewed on Day 0 as part of the enrollment process. Pregnancy test results for women of childbearing potential must be confirmed as negative prior to enrollment on Day 0. Day 0 evaluations prior to the vaccination are the baseline for subsequent safety assessments.

On Day 0, the subject will receive a single rAd5 vaccination at 10^{10} PU administered IM with a needle and syringe or Biojector (according to the randomization assignment) in the deltoid muscle of the arm. Following study injection, the subject will be observed for a minimum of 30 minutes. Vital signs (temperature, blood pressure, pulse and respiratory rate) will be recorded between 30 and 45 minutes post-injection and the injection site will be inspected for evidence of local reaction. In keeping with the NIH Clinical Center policy and good medical practice, acute medical care will be provided to subjects for any immediate allergic reactions or other injury resulting from participation in this research study.

Subjects will be given a "Diary Card," to use as a memory aid, for documentation of temperature and symptoms daily for 5 days. For subjects with internet access this may be done through a secure database. Subjects will be trained to utilize the secure database or complete the hard copy diary card depending on their preference. Either the subject entered electronic record or the written diary card may be used as a source document. When neither a written nor electronic diary is available from the subject, the study clinician will note the source of reactogenicity information recorded in the study database.

The first follow-up will be performed by telephone on the second day (±1 day) following the rAd5 injection. A clinic visit will occur if indicated by the telephone interview. Events reported in the telephone interview that will require a clinic visit include rash, urticaria (hives), fever of 38.7°C (Grade 2) or higher, unusual local reactogenicity or significant impairment in activities of daily living (ADL). Diary Card data will be reviewed with the subject at the follow-up visit after completion. At study day 7 (±1 day) following the rAd5 vaccination, study subjects will be evaluated at a clinic visit. Erythema, induration or skin lesions will be documented by measurement of perpendicular diameters. A photograph of the vaccination site will be taken if there are any clinical findings. When photos are taken, clinic staff will tag with metric ruler, subject number, date, and visit identifiers placed below the vaccination site prior to photographing.

Subjects will also be interviewed at the final clinical visit regarding social harms, including problems with employment, travel, immigration, access to insurance, medical or dental care, and negative reactions from family, friends, and co-workers.

Stored samples for immunological studies will be collected at timepoints shown in Appendix III. For Group 2 subjects, the PBMC for immunological analysis at Week 4 (-3 to +7 days) will be collected by apheresis, unless the subject is unwilling, ineligible by the NIH Clinical Center Department of Transfusion Medicine (DTM) Apheresis Clinic standards, or is unable to get a suitable appointment. To be eligible for apheresis, the hemoglobin value at Week 2 must be ≥11.5 g/dL, and the subject must meet DTM criteria for a research apheresis procedure on the day of apheresis. If apheresis is not done, PBMCs will be obtained from 80 mL of blood collected by standard phlebotomy. Each apheresis procedure will be carried out by trained DTM medical staff using automated cell separator devices. Whole blood is withdrawn from a venipuncture site in an antecubital vein at a rate of 50 to 80 mL/min and directed into the cell separator, where cellular and plasma fractions are separated by centrifugation. Mononuclear cells are harvested into a component bag, and the remaining red cells, platelets and plasma are returned to the subject via the same vein. Anticoagulation is achieved using citrate (ACD-A) or equivalent at a whole blood to anticoagulant ratio of 12:1. Maximum extracorporeal blood volume during the procedure ranges from 400 mL to 800 mL, depending on the subject's hematocrit. These procedures are carried out in such a way that the extracorporeal volume is no more than 15% of the blood volume in adults and these volumes are calculated using standard formulae in the DTM Apheresis Clinic. In this study, the procedure will usually involve two to four discontinuous-flow passes. Each pass takes about 30 minutes, processes about 500 mL of whole blood, and yields about 0.5×10^9 mononuclear cells. Thus, one to two hours are required to process 1 to 2 liters of blood and obtain about 1×10^9 to 2×10^9 leukocytes. The cell differential in the product is about 65% lymphocytes, 20% monocytes and the remainder granulocytes. The packed red cell loss during the procedure is about 20 mL.

Refer to the table in Appendix III for details on schedule of evaluations and the window permitted for completion. After Day 0, the deviations from the visit windows are discouraged and will be recorded as protocol deviations, but are permitted, at the discretion of the PI (or designee) in the interest of obtaining subject safety and immunogenicity evaluations following exposure to the investigational vaccine. Study evaluations include the following:

- "VRC 015 Assessment of Understanding" Quiz
- Signature of study participation informed consent form for VRC 015

- Clinical evaluations: vital signs and weight; targeted physical exam on any visit if indicated by interim complaints or laboratory findings.
- Interim medical history.
- Counseling on HIV and avoidance of pregnancy
- Study vaccination.
- Post-injection vital signs and assessment of injection site at 30 to 45 minutes after a study vaccination.
- Diary Card: Baseline on day of vaccination; 5-day diary card for self-assessment by subject following each vaccination. The diary card will include the parameters: unusually tired/feeling unwell, muscles aches (at other than injection site), headache, chills, nausea, and pain/tenderness at injection site. Subjects will also record highest measured temperature, measurement of perpendicular diameters for redness and swelling at injection site and note if there is evidence of a skin lesion at the vaccination site. The diary cards are collected at the earliest clinic visit after each injection when the card is complete, mailed into the clinic, or in real-time by secure web-based subject data entry system, depending on the preference of subject.
- Serum or urine pregnancy test, for females of reproductive potential.
- HLA (human leukocyte antigen) type: may be done from a sample collected at any timepoint, (if results available from prior testing these may be used).
- CBC, differential, platelet count.
- Creatinine and ALT.
- HIV testing: ELISA (also Western blot if ELISA is positive) and HIV PCR.
- HIV specific antibody research assays: The assays will not be performed immediately, but rather completed at a later date using frozen samples. Additional timepoints using stored samples may be performed if of interest.
- ELISpot and Intracellular cytokine staining (ICS) assays: The assays will not be performed immediately, but rather completed at a later date using frozen samples. PBMC and plasma for storage will be saved from the blood collected for these assays. When PBMC are collected by apheresis, a plasma sample will be obtained from a tube collected prior to starting the apheresis. PBMC may also be used for epitope mapping and other exploratory immunological studies. Other immunological assays may also be performed from stored samples.
- Social Impact Questionnaire: The Social Impact questionnaire will include parameters: personal relationships, travel or immigration, employment, education, medical or dental, health insurance, life insurance, housing, military/other government agency and other. It is a required evaluation at the last study visit but may be completed on any visit in which the subject indicates that he or she has experienced a social impact.
- Serum for archiving.
- Adenovirus Serology: This assay may be done from archive samples and does not require a

separate blood collection.

4.2.3 Long-term Follow-up:

After the Week 24 clinic visit, subjects will be contacted and encouraged to return for a clinic visit at Week 76 (± 28 days), Week 128 (± 28 days), Week 180 (± 28 days) and Week 232 (± 28 days). Subjects will be interviewed about the following:

- interval life-threatening adverse events;
- persistent or significant disability/incapacity;
- non-elective hospitalizations;
- new chronic diseases requiring ongoing medical management or medication (including HIV infection);
- outcomes of any pregnancies (including if there were any congenital anomalies/birth defects)

When completed as a clinic visit, HIV testing (ELISA with Western blot if positive and HIV PCR) and a research immunology blood draw (PBMC, plasma and serum) may also be completed. A subject may opt to be contacted (e.g., by telephone or mail) rather than have a clinic visit. If there are any subject deaths in the interval between Week 24 and Week 232, an attempt will be made to obtain information about cause of death. Subjects may also be contacted at other times to confirm contact information and to provide new information about study results.

4.3 MONITORING FOR HIV INFECTION

It is possible that this vaccination regimen will induce immunologic responses that are detected by standard HIV screening techniques, even though the vaccine will not cause HIV infection. The following steps will be taken to ensure detection of HIV infection and to protect participants from adverse consequences associated with an HIV antibody test that indicates a positive antibody response to the vaccine:

- Study participants will receive regularly scheduled counseling regarding avoidance of HIV infection in accordance with the most recent Centers for Disease Control and Prevention HIV Counseling Guidelines.

- Study participants will be screened for HIV infection periodically while participating in the study (see Appendix III for schedule of testing).

- If there is any clinical or laboratory indication of HIV infection, any test required to make a definitive diagnosis, including Western blot analysis, viral load measurement (PCR), or other tests will be performed.

- Confirming tests will be performed as soon as possible once a positive antibody response is identified. Participants will be promptly informed if they are HIV-infected. Participants who are found to have vaccine-induced antibody responses, but with no evidence of HIV infection, will be informed that they are not HIV-infected. Written documentation describing any vaccine-induced antibody response and confirming data will be provided when the study is completed. This should be sufficient evidence that the antibody response as of the date of testing resulted from vaccination and not from naturally occurring infection. Participants

with vaccine-induced antibody will be provided with the opportunity for HIV antibody testing at the VRC Clinic to monitor their serological status. This may be done through extra visits during the long-term follow-up for this study or through a VRC sample collection protocol. Participants will be counseled regarding the potential for antibody responses and the implications of such responses prior to participation in the study.

4.4 Intercurrent HIV Infection

The vaccine cannot cause HIV infection. Subjects who become HIV infected due to other causes while participating in the study will be referred for their medical care and treatment and management of the disease. They may be given the opportunity to enroll in an appropriate study of acute HIV infection or a long-term follow-up study, if one is available. The NIH investigators will not be responsible for providing ongoing medical care or antiretroviral medications in the event of HIV-1 infection.

4.5 Concomitant Medications

Concomitant medications are recorded at screening and every study visit. If an enrolled subject develops the need for a medication that is prohibited by the eligibility criteria, then further study injections will be discontinued. If an FDA-approved live attenuated vaccine is required during the study vaccination schedule for an immediate medical need, then study injections must be discontinued. If an FDA-approved subunit or killed vaccine is required for an immediate medical need, then it must be given at least 14 days before or 14 days after the study injection. If it will not imperil a subject's health, FDA-approved vaccines should be deferred until at least 28 days after the study injection. Any subject who receives the study injection will continue with the clinical and laboratory evaluations specified by the study through the full follow-up period.

4.6 Criteria for Withdrawal of a Subject from Study Participation

In VRC 015 there is only one administration of the study agent, which occurs on the day of enrollment. Once a subject has received the study agent completion of all follow-up visits will be encouraged. However, subjects may choose to discontinue study participation or a subject may be discontinued from participation by the Principal Investigator for repeated failure to comply with protocol requirements or if the IRB, FDA, or study sponsor require that all study visits be discontinued.

4.7 Criteria for Pausing the Study and Resuming the Study

The Principal Investigator will closely monitor and analyze study data as they become available and will make determinations regarding the presence and severity of adverse events. The DAIDS Medical Officer will provide an independent review of adverse events that have a bearing on study pauses. The administration of study injections and new enrollments will be paused and the investigational new drug application (IND) sponsor will be promptly notified according to the criteria that follow:

- <u>One</u> (or more) subject experiences a Grade 4 or Grade 5 adverse event that is assessed as possibly, probably or definitely related to a study vaccine, or

- <u>Two</u> (or more) subjects experience the same Grade 3 adverse event assessed as possibly, probably or definitely related to a study vaccine (except that Grade 3 solicited reactogenicity

symptoms of injection site pain/tenderness, fever, fatigue/malaise, myalgia, chills, headache or nausea will not result in a study pause).

Plan for Review of Pauses and Resuming Rules:

The study injections and enrollments would resume only if review of the adverse events that caused the pause resulted in a recommendation to permit further study injections and study enrollments. The reviews to make this decision will occur as follows:

The IND Sponsor, in consultation with the Principal Investigator, will conduct the review and make the decision to resume or close the study for the Grade 3 events that meet the criteria for pausing the study. As part of the pause review, the reviewers will also advise on whether the study needs to be paused again for any subsequent Grade 3 event of the same type.

The IND Sponsor, with participation by the Principal Investigator, will consult with the FDA to conduct the review and make the decision to resume or close the study for any Grade 4 and Grade 5 adverse events that meet the criteria for pausing the study.

Safety data reports and changes in study status are submitted to the IRB promptly in accordance with Section 5.4 and institutional policy.

5. SAFETY AND ADVERSE EVENT REPORTING

5.1 ADVERSE EVENTS

An adverse event (AE) is any unfavorable or unintended change in body structure, body function or laboratory result associated temporally with the use of study treatment, whether or not considered related to the study treatment. Each adverse event will be graded according to the Division of AIDS Table for Grading Severity of Adverse Events (see Appendix IV). Because this is an HIV vaccine study, any HIV infection AEs will be tabulated on a separate table and recorded without requiring an investigator attribution ("relatedness to study agent"); severity will be assessed using the DAIDS Table guidance for estimating severity grade for event types not otherwise specified on the table.

5.2 SERIOUS ADVERSE EVENTS (SAE)

The term "Serious Adverse Drug Experience" is defined in 21 CFR 312.32 as follows: "Any adverse drug experience occurring at any dose that results in any of the following outcomes: Death, a life-threatening adverse drug experience, inpatient hospitalization or prolongation of existing hospitalization, a persistent or significant disability/incapacity, or a congenital anomaly/birth defect. Important medical events that may not result in death, be life-threatening, or require hospitalization may be considered a serious adverse drug experience when, based upon appropriate medical judgment, they may jeopardize the subject or require medical or surgical intervention to prevent one of the outcomes listed in this definition. Examples of such medical events include allergic bronchospasm requiring intensive treatment, blood dyscrasias or convulsions that do not result in inpatient hospitalization, or the development of drug dependency or drug abuse."

"Life-threatening" refers to an adverse event that at occurrence represents an immediate risk of death to the subject. An event that may cause death if it occurs in a more severe form is not considered life-threatening. Similarly, a hospital admission for an elective procedure is not

considered a Serious Adverse Event.

In Section 5.3 the term "Expedited Adverse Event" (EAE) encompasses the events that would be considered an SAE by the 21 CFR 312.32 definition.

5.3 Adverse Event Reporting to the IND Sponsor

The IND Sponsor, DAIDS, issued Version 2.0 of its EAE Manual in January 2010. For this study, the requirements that applied under Version 1.0 were in effect through the last required clinic visit (Week 24) and the updated Version 2.0 requirements are in effect for the Long-term Follow-up contacts.

All versions of the DAIDS EAE Manual are available on the Regulatory Support Center (RSC) website: http://rsc.tech-res.com/safetyandpharmacovigilance/.

Regardless of when they occur, AEs reported on an expedited basis must be documented through the DAIDS Adverse Event Reporting System (DAERS) by electronic submission within 3 reporting days of site awareness of the event. Access is available for authorized clinical staff through the RSC website: http://rsc.tech-res.com/safetyandpharmacovigilance/. RSC contact information is provided in Appendix II.

5.3.1 Reporting to the IND Sponsor through Study Week 24

Information on adverse events (AEs) is collected by Study Nurses and other clinic staff and entered into a computer database. The Principal Investigator and the Study Coordinator review these data on an ongoing basis.

The EAE requirements and definitions for this study through Study Week 24 for expedited reporting of adverse events (AEs) to the DAIDS RSC Safety Office are defined in "The Manual for Expedited Reporting of Adverse Events to DAIDS" (DAIDS EAE Manual) Version 1.0, dated May 6, 2004

EAE Reporting Level:

Study visits through Week 24 use the Standard Level of expedited AE reporting as defined in the DAIDS EAE Manual, Version 1.0 (May 6, 2004). Briefly summarized, Standard Level reporting requires completion of an EAE report form for the following types of AEs occurring after exposure to the study agent:

- Result in death regardless of relationship to study agent.
- Are congenital anomalies, birth defects, or fetal losses regardless of relationship to study agent.
- Result in persistent or significant disabilities or incapacities regardless of relationship to study agent.
- Are a suspected adverse drug reaction (i.e., definitely, probably, possibly, or probably not related to study agent) that requires hospitalization, or prolongs existing hospitalization OR requires intervention to prevent significant/permanent disability or death.
- Are life-threatening (including all Grade 4 adverse events) suspected adverse drug reactions (i.e., assessed as definitely, probably, possibly or probably not related to study

agent).

In addition, any event, regardless of grade, which in the judgment of a site investigator represents a serious adverse event, may be reported to the IND sponsor as an expedited report.

EAE Reporting Period:

AEs must be reported on an expedited basis at the Standard Level during the protocol-defined EAE Reporting Period, which for this study is from study enrollment until the last required clinical visit at Week 24 or until discontinuation of the subject from study participation for any reason.

After the end of the protocol-defined EAE reporting period stated above, the site must report serious, unexpected, clinical suspected adverse vaccine reactions if the study site staff becomes aware of the event on a passive basis, i.e. from publicly available information.

Study Agent for Expedited Reporting to DAIDS:

The study agent that must be considered when determining relationships of AEs requiring expedited reporting to DAIDS is VRC-HIVADV014-00-VP.

Grading Severity of Events:

The table for grading the severity of adverse events is: "The Division of AIDS Table for Grading the Severity of Adult and Pediatric Adverse Events, Version 1.0, Dec 2004 (with Clarification August 2009)" (see Appendix IV).

The IND sponsor is responsible for submitting IND safety reports to the FDA, as necessary, per 21 CFR 312.32. DAIDS submits IND safety reports as soon as possible, but no later than 15 days after initial receipt of the information.

5.3.2 Reporting to the IND Sponsor During Long-term Follow-up

The reporting guidelines in the updated DAIDS EAE Manual, Version 2.0, dated January 2010 apply to Part II subjects starting with the long-term follow-up contacts. For convenience the criteria from the EAE Manual for reporting an AE as a serious adverse event (SAE) are provided in the box below. However, the Manual should be consulted as well for further detail about reporting procedures to be used. Also ensure that any other protocol-specific reporting requirements are met.

Of special note, HIV infection is within the definition of events that need to be recorded during the long-term follow-up period in accordance with protocol **Section 4.2.3**; however, a new diagnosis of HIV infection will not be reported as an EAE, but will be recorded in an HIV diagnosis case report format without a severity grade or attribution assessment.

EAE Reporting Criteria During Long-term follow-up:

Complete an EAE report form for the following adverse events regardless of relationship to study agent:

- Results in death
- Is life-threatening[1]
- Requires inpatient hospitalization or prolongation of hospitalization[2]
- Results in persistent or significant disabilities/incapacity.
- Is a congenital anomaly/birth defect[3]
- Is an important medical event (may jeopardize the patient or may require intervention to prevent one of the outcomes above)

[1] "Life-threatening" refers to an event in which the patient was at risk of death at the time of the event. It does NOT refer to an event that hypothetically might have caused death if it were more severe.

[2] Per ICH SAE definition, hospitalization is NOT an adverse event (AE), but is an outcome of the event. **DO NOT REPORT**: Any admission unrelated to an AE (e.g., for labor/delivery, cosmetic surgery, administrative or social admission for temporary placement for lack of a place to sleep); protocol-specified admission (e.g., for a procedure required by protocol); admission for diagnosis or therapy of a condition that existed before receipt of study agent(s) **and** has not increased in severity or frequency as judged by the clinical investigator. (**NOTE**: A new AIDS-defining event in a subject already known to be HIV-infected would be considered an increase in severity of a pre-existing condition [HIV infection] and **would be** reportable.)

[3] Clinically insignificant physical findings at births including those regarded as normal variants do NOT meet reporting criteria. If a clinically significant anomaly is reported, all findings (including those of no individual significance) should be included in the same report. For example, do NOT report an isolated finding of polydactyly (extra fingers or toes) or Mongolian spot in an infant. But if either finding occurred with a major cardiac defect, report all findings in the SAE Report.

5.3.3 Attribution Categories

Consistent with the EAE Manual, Version 1.0 attribution categories used (i.e. terms used for assessment of relationship of AE to study agent) for reporting AEs to DAIDS through end of the clinical visits are:

- **Definitely Related**. The adverse event and administration of study agent are related in time, and a direct association can be demonstrated.

- **Probably Related**. The adverse event and administration of study agent are reasonably related in time, and the adverse event is more likely explained by study agent than other causes.

- **Possibly Related**. The adverse event and administration of study agent are reasonably related in time, and the adverse event can be explained equally well by causes other than study agent.

- **Probably Not Related**. A potential relationship between study agent and the adverse event could exist (i.e., the possibility cannot be excluded), but the adverse event is most likely explained by causes other than the study agent.

- **Not Related**. The adverse event is clearly explained by another cause not related to the study agent.

Under EAE Manual, Version 2.0 only two attribution categories apply as follows:

- **Related** – There is a reasonable possibility that the AE may be related to the study agent(s).

- **Not Related** – There is not a reasonable possibility that the AE is related to the study agent(s).

In this protocol, the "Definitely, Probably and Possibly" attributions used under EAE Manual, Version 1.0 are considered to map to the "Related" category under EAE Manual, Version 2.0, while the "Probably Not Related and "Not Related" attributions used under EAE Manual, Version 1.0 are considered to both map to the "Not Related" category under EAE Manual, Version 2.0.

5.4 REPORTING TO THE INSTITUTIONAL REVIEW BOARD

5.4.1 Adverse Event Reporting to the NIAID IRB

Adverse event reporting requirements to the NIAID IRB for this protocol are as follows:

- Investigators will submit a completed serious adverse event report to the NIAID IRB within 7 days after becoming aware of a subject death, a potentially life-threatening (grade 4) adverse event, an inpatient hospitalization (other than for elective reasons), a persistent or significant disability/incapacity, or a congenital anomaly/birth defect.

- Investigators will report within 15 days on any other event or condition regardless of grade, which in their judgment represents an event reportable to the IRB.

- Investigators will forward all IND safety reports and related FDA communications to the IRB within 15 days of receipt.

- A summary of all adverse events will be reported to the NIAID IRB with the annual submission of a request for continuing review.

5.4.2 Unanticipated Problem Reporting to the NIAID IRB

Unanticipated Problem reporting to the NIAID IRB is based upon the OHRP 2007 guidance (http://www.hhs.gov/ohrp/policy/advevntguid.html) and is defined as any incident, experience, or outcome that meets all three of the following criteria:

- unexpected in nature, severity, or frequency in relation to the research risks that are described in the protocol, informed consent, Investigator's Brochure, other study documents or in consideration of the characteristics of the subject population being studied; **and**
- is related to participation in the research; **and**
- places subjects or others at a greater risk of harm (including physical, psychological, economic, or social harm) than was previously known or recognized.

Unanticipated problems meeting these criteria will be reported to the IRB, within 7 days of investigator awareness.

5.4.3 Protocol Violation Reporting to the NIAID IRB

A Protocol Violation is defined as any change, divergence, or departure from the study procedures in an IRB-approved research protocol that meets the criteria for expedited reporting described on the IRB's Protocol Violation Form and has a major impact on the subject's rights, safety, or well-being and/or the completeness, accuracy or reliability of the study data.

The Investigator will report, within 7 days of awareness, any Protocol Violation that meets the IRB's expedited reporting criteria. A summary of all protocol violations will be reported to the NIAID IRB with the annual submission of a request for continuing review.

5.5 Serious Adverse Event Reporting to the Institutional Biosafety Committee

The Institutional Biosafety Committee (IBC) (Building 13, Room 3K04, NOH, Bethesda, MD) has a responsibility to review research using recombinant DNA for compliance with NIH Guidelines. In keeping with IBC requirements, any SAE reports sent to the IRB will be provided to the IBC at the same time.

6. STATISTICAL CONSIDERATIONS

6.1 Overview

This study is a single-center, randomized trial to assess the safety and tolerability of an rAd5 HIV vaccine in HIV-uninfected adults. A preliminary assessment of immunogenicity will also be performed.

6.2 Objectives

The primary objective is to evaluate the safety and tolerability in humans of the vaccination administered by Biojector. Secondary objectives include evaluating the immunogenicity of the vaccination when administered by Biojector, the development of adenovirus serotype 5 neutralizing antibody and to monitor the social impact of participating in an HIV-1 vaccine trial.

6.2.1 Safety Endpoints

Assessment of product safety will include clinical observation and monitoring of hematological and chemical parameters. Safety will be closely monitored after injection and evaluated through 24 weeks following the study vaccination. See Section 4.2 and Appendix III for details and specified time points. The following parameters will be assessed:

- Local reactogenicity signs and symptoms
- Systemic reactogenicity signs and symptoms

- Laboratory measures of safety
- Adverse and serious adverse experiences

6.2.2 Immunogenicity Endpoints

The principal immunogenicity endpoints for cellular immune responses are measured at 4 weeks after the rAd5 vaccination. They will consist of HIV-1-specific T cell responses, as measured by ELISpot and intracellular cytokine staining (ICS) assays and by research ELISA for vaccine-specific antigens. These and other immunogenicity assays will be performed at other study timepoints as exploratory evaluations.

6.3 Sample Size and Accrual

Recruitment will target healthy, HIV-uninfected adult subjects. Group 1 subjects will be 18 through 50 years old and HIV vaccine-naïve. Group 2 subjects will be 18 through 55 years old and must be former subjects from another trial in which they received at least one injection with the VRC rAd5 candidate vaccine. The accrual target is 20 subjects in Group 1 and 10 to 20 subjects in Group 2. Each group will be randomized equally to administration of the vaccine by Biojector or by needle injection. The required clinical follow-up is through Study Week 24. As Group 2 is restricted to a small number of potential subjects, the statistical assessment of safety and immunogenicity data for this group will be limited by the number of actual enrollments.

6.3.1 Randomization of Treatment Assignments

Subjects will be simultaneously enrolled into Group 1 and Group 2 as they are found to be both eligible and available to begin participation. The randomization sequence will be obtained by computer-generated random numbers and provided by the Protocol Statistician to the Pharmacist and Data Management Center prior to study initiation. The study number is assigned to a subject through completion of the eligibility checklist in the electronic study database and will remain blinded to VRC Clinic staff and subjects until the enrollment confirmation is generated by the study database, at which time the assignment is permitted to be known to all. Study ID numbers 015001 through 015020 will be reserved for Group 1, which is limited to 20 subjects. Because Group 1 is designed to include at least 8 with low Ad5Ab titer, 8 with high Ad5Ab titer and allows 4 with any titer, the ID numbers are further linked to the Ad5Ab titer obtained at screening as shown in Table 6.1. The randomization plan will ensure that each column of numbers will have 1:1 randomization to N/S or Biojector for that study injection.

Table 6.1 Study ID Numbers for Group 1

Low Ad5Ab	High Ad5Ab	Any Ad5Ab
015001	015009	015017
015002	015010	015018
015003	015011	015019
015004	015012	015020
015005	015013	
015006	015014	
015007	015015	
015008	015016	

Study ID numbers 015021 through 015040 will be reserved for Group 2, which may have up to 20 subjects. In Group 2 ID numbers will be sequentially assigned and the randomization plan will have 1:1 randomization to N/S or Biojector for the study injection. This will be achieved using permuted block randomization, with random block sizes for the first 16 people in Group 1 (blocks of size 4 or 8), a block of size 4 for the remaining people in Group 1, and blocks of size 4 or 6 chosen randomly for Group 2.

6.3.2 Sample Size Considerations for Safety

The goal of the safety evaluation for this study is to identify safety concerns associated with injection by two different injection devices. Sample size calculations for safety are expressed in terms of the ability to detect serious adverse events. The sample size in subgroups 1a and 1b is expected to be exactly 10 per subgroup. The sample size in subgroups 2a and 2b is expected to be at least 5 but may be up to 10 per subgroup. The discussion below is based on 10 per subgroup.

The ability of the study to identify serious adverse events is best expressed by the maximum true rate of SAE that would unlikely to be observed and the minimum true SAE rate that would very likely be observed. Probabilities of observing 0 or 2 or more events are presented in Table 6.2 for a range of possible true SAE rates. These calculations provide a complete picture of the sensitivity of this study design to identify potential safety problems with the rAd5 vaccine. For example, if the true SAE rate is .01, then there is a probability of .904 of observing no events and a probability of .004 of observing two or more events among the 10 vaccinees in any subgroup. Table 6.3 gives the exact 95% confidence intervals for any observed number of adverse events out of any group of size 10.

Table 6.2: Probability of SAE or immune response for different safety and immunogenicity scenarios

	Within each group of size 10	
True event rate	Pr(0 observed event)	Pr(2 or more observed events)
0.01	0.904	0.004
0.03	0.817	0.016
0.05	0.599	0.086
0.1	0.349	0.264
0.2	0.107	0.624
0.3	0.028	0.851
0.4	0.006	0.954
0.5	<0.001	0.989
0.6	<0.001	0.998
0.7	<0.001	>.999

Table 6.3: 95% Confidence intervals for all possible observed rates within each subgroup of size 10

Observed rates	95% confidence interval	
	Lower bound	Upper bound
0/10	0	0.309
1/10	0.003	0.445
2/10	0.025	0.556
3/10	0.067	0.653
4/10	0.122	0.738
5/10	0.187	0.813
6/10	0.262	0.878
7/10	0.348	0.933
8/10	0.444	0.975
9/10	0.555	0.998
10/10	0.692	1

6.3.3 Sample Size Considerations for Immunogenicity

While the immunogenicity of this vaccine among the subgroups is a secondary objective, it is nonetheless useful to summarize the information about immunogenicity that will be available from this study.

Table 6.3 is also applicable for the evaluation of immunogenicity in terms of immune response rates. It gives the exact 95% confidence intervals for any possible number of observed responses. For example, if we observe 7 responses out of the 10 vaccinees within a subgroup then the 95% exact binomial confidence interval for the true response rate will range from .348 to .933.

The bottom part of Table 6.2 gives the probabilities of observing a small number of responses (0, 1, or at least 2) for a range of true underlying response rates. For example, if the true response rate is .6, then there is a probability of .998 of observing at least two immune responses and a probability of 0.9999 of observing at least one immune response among the 10 vaccinees in any subgroup.

6.4 STATISTICAL ANALYSIS

Since enrollment is concurrent with receiving the first study vaccination it is expected that all subjects will provide some safety data.

All statistical analyses will be performed using SAS, R and S-Plus statistical software.

No formal multiple comparison adjustments will be employed for safety endpoints or secondary endpoints.

6.4.1 Analysis Variables

The analysis variables consist of baseline variables, safety variables, immunogenicity and social impact variables for primary and secondary objective analyses.

6.4.2 Baseline Demographics

Baseline characteristics including demographics and laboratory measurements will be summarized using descriptive statistics.

6.4.3 Safety Analysis

Summaries of the number and percentage of subjects experiencing any AE or reactogenicity will be tallied by subgroup, and presented along with exact 95% confidence intervals for the proportion.

Reactogenicities:
The number and percentage of subjects experiencing each type of reactogenicity sign or symptom will be tabulated by severity. For a given sign or symptom, each subject's reactogenicity will be counted once under the maximum severity for all assessments.

Adverse Experiences:
Adverse experiences (AEs) are coded into MedDRA preferred terms. The number and percentages of participants experiencing each specific adverse event will be tabulated by severity and relationship to treatment. For the calculations in these tables, each participant's adverse experience will be counted once under the maximum severity or strongest recorded causal relationship to treatment.

A complete listing of adverse experiences for each participant will provide details including severity, relationship to treatment type, onset, duration and outcome.

Local laboratory values:
Boxplots of local laboratory values will be generated for baseline values and for values measured during the course of the study. Each boxplot will show the 1st quartile, the median, and the 3rd quartile. Outliers, or values outside the boxplot, will also be plotted. If appropriate, horizontal lines representing boundaries for abnormal values will be plotted.

6.4.4 Immunogenicity Analysis

For the analysis of the secondary immunogenicity endpoints, the proportion of participants in each subgroup who produce a response at the specified timepoint will be calculated, along with exact 95% confidence interval estimates for these proportions. The statistical analysis for immunogenicity will employ the intent-to-treat principle, i.e., all data from enrolled participants will be used. The only exception will be to exclude data from HIV-infected participants at or post infection. If the HIV positivity status of an infected participant is unknown at the time that the first sample for immunogenicity assessments is drawn, then all data from that participant will be excluded from the analysis.

For exploratory endpoints, similar analyses will be conducted. If assay data are qualitative (i.e., positive or negative) then analyses will be performed by tabulating the frequency of positive response for each assay at each time point that an assessment is performed. Binomial response rates will be presented with their corresponding exact 95% confidence interval estimates.

For the underlying continuous or count-type outcomes, graphical and tabular summaries of the underlying distributions will be made. These summaries may be performed on transformed data (e.g., log transformation) to better satisfy assumptions of symmetry and homoscedasticity.

Missing responses will be assumed to be missing at random, i.e., conditional on the observed data the missingness is independent of the unobserved responses. Graphical descriptions of the longitudinal immune responses will also be given. Regression modeling or analysis of

covariance may be used to examine the effect of Ad5Ab or other differences such as any differences in immunogenicity endpoints associated with leukapheresis.

6.4.5 Interim Analyses

Interim analyses of immunogenicity in Group 1 may be performed for ELISpot and ICS assays once all subjects are at least 4 weeks after the study vaccination; interim analyses of Group 2 immunogenicity may be performed as data accumulate. The purpose of the reports is to provide basic immunogenicity data to inform those who are making future clinical trial development-related decisions in a timely manner. The results of this interim immunogenicity analysis will not influence the conduct of the VRC 015 trial in terms of early termination or completion of later safety or immunogenicity endpoint assessments.

7. PHARMACY PROCEDURES

The study Groups and vaccination schedule are shown in Table 4.1. Refer to the Investigator's Brochure for further information about study products.

7.1 STUDY PRODUCTS AND INJECTION REGIMEN

The study includes an investigational adenoviral vector vaccine as follows:

- rAd5 1 x 10^{10} PU/mL: VRC-HIVADV014-00-VP (multiclade HIV-1 recombinant adenovirus-5 vaccine, rAd5 vaccine)

Group 1

Group 1a: VRC- HIVADV014-00-VP (rAd5) 1 x 10^{10} PU administered as 1 mL IM by needle and syringe in either deltoid at Day 0

Group 1b: VRC- HIVADV014-00-VP (rAd5) 1 x 10^{10} PU administered as 1 mL IM by Biojector in either deltoid at Day 0

Group 2

Group 2a: VRC- HIVADV014-00-VP (rAd5) 1 x 10^{10} PU administered as 1 mL IM by needle and syringe in either deltoid at Day 0

Group 2b: VRC- HIVADV014-00-VP (rAd5) 1 x 10^{10} PU administered as 1 mL IM by Biojector in either deltoid at Day 0

7.2 STUDY AGENT STORAGE, SHIPPING AND PRESENTATION

7.2.1 Study Agent Storage

Refer to the vial label for storage temperature information. The site pharmacist must report any storage temperature excursions promptly to the IND sponsor's authorized representative. The product must be quarantined in a separate area. The IND Sponsor's authorized representative will notify the site pharmacist if continued clinical use of the product is acceptable.

7.2.2 VRC-HIVADV014-00-VP Adenoviral Vector Vaccine, rAd5

The adenoviral vector vaccine is supplied in a 3 mL glass vial containing a clear colorless isotonic sterile solution. Each vial contains 1.2 mL ± 0.1 mL volume with 1 x 10^{10} PU/mL. Each

vial contains 20% (mg) over the amount to be injected. The rAd5 vaccine is formulated with Final Formulation Buffer (FFB).

7.3 PREPARATION OF STUDY AGENT FOR ADMINISTRATION

This section describes how the site pharmacist will prepare study injections. Clinician instructions on how to select an arm and administer the injections are in Section 4.2.2.

7.3.1 VRC-HIVADV014-00-VP Adenoviral Vector Vaccine: Preparation for Administration

To prepare, remove a vial of rAd5 vaccine from the freezer and allow to equilibrate to room temperature. Using aseptic technique, withdraw 1 mL of the rAd5 vaccine into a 3 or 5 mL syringe. Vials are for single use only and should not be refrozen after thawing.

- One 1 mL injection of the 1 x 10^{10} PU/mL preparation will be administered for each 10^{10} PU dose. Injections will be administered IM into deltoid muscle by the method indicated in the randomization plan and must be given within 4 hours of removing the vaccine vial from the freezer.

7.3.2 Administration of VRC-HIVADV014-00-VP by Needle and Syringe

When the randomization assignment includes IM injection by needle and syringe of the rAd5 vaccine (Groups 1a and 2a), an individual syringe with 10^{10} PU in a 1 mL volume will be prepared by the Clinical Center pharmacy and labeled with the subject identifier for transport to the clinic. The pharmacy will also label with information about date and time after which the preparation may not be used. When preparing a dose in a syringe for administration, consideration should be given to the volume of solution that may remain in the needle after the dose is administered.

The clinician administering the injection will select a needle of appropriate gauge and length for IM injection. For IM injections the recommendation is to use a 21-gauge needle, with a length of 1 or 1.5 inch (depending on subject arm size) in order to ensure intramuscular injection into the deltoid muscle.

7.3.3 Administration of VRC-HIVADV014-00-VP by Biojector

When the randomization assignment includes IM injection by Biojector of the rAd5 vaccine (Groups 1b and 2b) the 10^{10} PU dose of vaccine will be prepared in the Clinical Center pharmacy and the prepared Biojector syringe labeled with the subject identifier will be delivered to the clinic for administration. The pharmacy will also label with information about date and time after which the preparation may not be used.

The Biojector® 2000 Needle-Free Injection Management System (Biojector) will be used as directed by the company (Bioject Medical Technologies Inc., Tualatin, Oregon). Biojector utilizes sterile, single-use syringes for administration of volume up to 1.0 mL. The study agent is delivered under pressure by a compressed CO_2 gas cartridge that is stored inside the Biojector. Neither the material being injected nor injection site skin preparation requires deviation from standard procedures. The CO_2 does not come in contact with the injectate and the syringe design prevents any back splatter or contamination of the device by tissue from the participant. This system has FDA clearance for delivering intramuscular injections of vaccine.

7.4 STUDY AGENT LABELING

Study agent vials will be individually labeled with the name of the material, dose, pH, volume, lot number, concentration, storage instructions, Investigational Use Statement ("Caution: New Drug – Limited by Federal Law to Investigational Use"), and manufacturer information.

7.5 STUDY AGENT ACCOUNTABILITY

The study pharmacist will be responsible for maintaining an accurate record of the codes, inventory, and an accountability record of vaccine supplies for this study. Electronic documentation as well as paper copies will be used.

7.6 STUDY AGENT DISPOSITION

The empty vials and the unused portion of a vial will be discarded in a biohazard containment bag and incinerated or autoclaved. Any unopened vials that remain at the end of the study will be returned to the production facility or discarded at the discretion of the sponsor in accordance with policies that apply to investigational agents. Partially used vials will not be administered to other subjects or used for *in vitro* experimental studies. They will be disposed of in accordance with institutional or pharmacy policy.

8. HUMAN SUBJECT PROTECTIONS AND ETHICAL OBLIGATIONS

This research study will be conducted in compliance with the protocol, Good Clinical Practices (GCP), and all applicable regulatory requirements.

8.1 INFORMED CONSENT

The study informed consent is provided in Appendix I. It describes the investigational product to be used and all aspects involved in protocol participation.

Before a subject's participation in the study, it is the investigator's responsibility to obtain written informed consent from the subject, after adequate explanation of the aims, methods, anticipated benefits, and potential hazards of the study and before any protocol-specific procedures or study medications are administered. The Assessment of Understanding quiz will be completed before the study consent is signed (see **Section 4.1**).

The acquisition of informed consent will be documented in the subject's medical records, as required by 21 CFR 312.62. The informed consent form will be signed and personally dated by the subject and the person who conducted the informed consent discussion. The original signed informed consent form will be retained in the medical chart and a copy will be provided to the subject.

8.2 RISKS AND BENEFITS

8.2.1 Risks

VRC-HIVADV014-00-VP: The adenoviral vector vaccine, VRC-HIVADV014-00-VP has previously been administered at the NIH Clinical Center in Phase I clinical trials at dosages up to 10^{11} PU per injection. Following injection, vaccinees may have a flu-like set of symptoms headache, muscle aches, malaise and chills (with or without fever) starting 12-16 hours after vaccination and lasting a few hours. Some of these symptoms may be moderate in severity. A

few subjects have had nausea. Some subjects have had injection site pain or discomfort in the first few days after a vaccination. These symptoms improved after treatment with over-the-counter medicine. A small proportion of subjects (about 5%) may have a self-limited grade 2 (>81 cm^2) injection site erythema reaction that starts a few days after injection and resolves without treatment.

The VRC-HIVADV014-00-VP vaccine has been administered to more than 140 subjects at different doses over the course of five different Phase I trials at the Vaccine Research Center, as well as to more than 800 subjects in international multicenter studies, but has not been delivered by Biojector prior to this study. The vaccine has been evaluated as safe and well tolerated by diverse investigators in the multicenter trials and reactogenicity has been mild to moderate. In a recent study, VRC 011, the rAd5 vaccine when administered intradermally, was found to sometimes cause similar subcutaneous lesions as those found in intramuscular administration of the DNA vaccine via Biojector. The skin lesions observed were superficial, about 1 cm in diameter. Scabs formed at the site following the vaccination and came off a few days later. The skin healed without treatment in a few weeks. This type of reaction has not been observed following IM needle injections of the rAd5 vaccine.

The effect of the study vaccine on a fetus or nursing baby is unknown, so female subjects of child bearing potential will be required to agree to use birth control for sexual intercourse beginning 21 days prior to enrollment and continuing through Week 24. Women who are pregnant or nursing will be excluded from the study.

This vaccine may cause a positive HIV antibody test using the standard screening test. A positive or indeterminate test may have a negative employment and social impact. Western blot analysis and HIV PCR or other testing will be done to either exclude or confirm HIV infection. ELISA, Western Blot, and PCR results will be discussed with the study subject as they become available.

Risks of Blood Drawing: Blood drawing may cause pain, bruising; may infrequently cause a feeling of lightheadedness or fainting, and rarely, may cause infection at the site where the blood is taken.

Risks of Apheresis: Certain adverse events during apheresis procedures are expected, such as vasovagal episodes (lightheadedness, dizziness, syncope, nausea, vomiting) related to needle insertion, and cutaneous paresthesias, chills, nausea, and heartburn caused by the citrate anticoagulant used during the procedure. Hematoma formation and transient cutaneous neurological complaints related to needle insertion might also be seen. These events occur in 1-2% of donations from healthy volunteer blood donors. Vasovagal reactions are handled by postural manipulation and fluid administration. Citrate reactions are usually relieved by slowing the rate of the anticoagulant infusion and by administering oral calcium carbonate tablets (such as Tums®). Rarely, machine malfunction may results in loss of as much as a single unit (500 mL) of whole blood.

Both as reported by others and as observed at the NIH Clinical Center, subjects may rarely sustain a drop in total lymphocyte count (and $CD4^+$ T cell count) when lymphapheresis is performed frequently over a short period of time. However, participants in this study will have no more than one apheresis procedure so this is not expected to occur following the single apheresis in this study.

Other Risks: Subjects may believe that this vaccine provides protection, and therefore practice riskier behavior. They will receive extensive counseling throughout the study to address this potential problem.

8.2.2 Benefits

It is unknown if any benefit will result from study participation. Others may benefit from knowledge gained in this study that may aid in the development of an HIV vaccine.

8.3 Institutional Review Board

A copy of the protocol, proposed informed consent form, other written subject information, and any proposed advertising material will be submitted to the IRB for written approval.

The investigator must submit and, where necessary, obtain approval from the IRB for all subsequent protocol amendments and changes to the informed consent document. The investigator will notify the IRB of deviations from the protocol and serious adverse events.

The investigator will be responsible for obtaining IRB approval of the annual Continuing Review throughout the duration of the study.

8.4 Protocol Registration

The Division of AIDS, NIAID is the IND sponsor for this protocol. Protocol registration must occur before subjects are enrolled in this study. The Institutional Review Board (IRB) must approve the protocol and consent form. The protocol must be submitted to the Institutional Biosafety Committee (IBC). Approval letters from both the IRB and IBC must be submitted to the Division of AIDS Regulatory Support Center (RSC) Protocol Registration Office (see Appendix II) with the initial protocol registration. Subsequent protocol amendments must also be registered with the RSC Protocol Registration Office (refer to the April 9, 2008 protocol registration procedures for VRC protocols conducted at the NIH Clinical Center). Protocol registration material can be sent electronically to epr@tech-res.com. For questions regarding protocol registration, please call (301) 897-1707 or email Protocol@tech-res.com.

8.5 Subject Confidentiality

The investigator must ensure that the subject's anonymity is maintained. Individual identifying information will not be included in any reports; subjects will be identified only by coded numbers. All records will be kept confidential to the extent provided by federal, state and local law. Medical records are made available for review when required by the Food and Drug Administration or other authorized users, such as the vaccine manufacturer, only under the guidelines set by the Federal Privacy Act. Direct access includes examining, analyzing, verifying, and reproducing any records and reports that are important to the evaluation of the study. The investigator is obligated to inform the subjects that the above named representatives will review their study-related records without violating the confidentiality of the subjects.

8.6 Plan for Use and Storage of Biological Samples

The June 12, 2006 memorandum "Research Use of Stored Human Samples, Specimens or Data" requires that all NIH IRB-approved protocols in which intramural research program researchers intend to collect and store human specimens or data must include a written description of the intended use of the samples; how they will be stored; how they will be tracked; what will happen

to these at the completion of the protocol, and what circumstances would prompt the PI to report to the IRB loss or destruction of samples. We will apply the specified provisions to the stored samples from this protocol as follows:

Intended use of the samples/specimens/data:
Samples, specimens and data collected under this protocol may be used to conduct protocol-related safety and immunogenicity evaluations, exploratory laboratory evaluations related to the type of infection the vaccine was designed to prevent, exploratory laboratory evaluations related to vaccine research in general and for research assay validation. Genetic testing may be performed in accordance with the genetic testing information that was included in the study informed consent.

How stored samples, specimens and data from sample use will be stored:
All of the stored study research samples are labeled by a code (such as a number) that only the VRC Clinic can link to the subject. Samples are stored at the NIAID Vaccine Immune T-Cell and Antibody Laboratory (NVITAL), Gaithersburg, MD or VRC Laboratories in Building 40, which are both secure facilities with limited access. Data will be kept in password-protected computers. Only investigators or their designees will have access to the samples and data.

How samples/specimens/data will be tracked:
Samples will be tracked in the Laboratory Information Management System (LIMS) database.

What will happen to the samples/specimens/data at the completion of the protocol:
In the future, other investigators (both at NIH and outside) may wish to study these samples and/or data. IRB approval must be sought prior to any sharing of samples. Any clinical information shared about those samples would similarly require prior IRB approval. The research use of stored, unlinked or unidentified samples may be exempt from the need for prospective IRB review and approval. Exemption requests will be submitted in writing to the NIH Office of Human Subjects Research, which is authorized to determine whether a research activity is exempt.

At the time of protocol termination, samples will remain in the NVITAL facility or VRC laboratories or, after IRB approval, transferred to another repository. Data will be archived by the VRC in compliance with requirements for retention of research records, or after IRB and study sponsor approval, it may be either destroyed or transferred to another repository.

Circumstances that would prompt the PI to report loss or destruction of samples/specimens/data to the IRB:
The NIH Intramural Protocol Violation definition related to loss of or destruction of samples will be followed in reporting to the IRB. Any loss or unanticipated destruction of samples (for example, due to freezer malfunction) or data (for example, misplacing a printout of data with identifiers) that compromises the scientific integrity of the study will be reported to the IRB. The PI will also notify the IRB if the decision is made to destroy the remaining samples.

8.7 SUBJECT IDENTIFICATION AND ENROLLMENT OF STUDY PARTICIPANTS

All study activities will be carried out at the Clinical Center at the National Institutes of Health.

Study subjects will be recruited through on-site and off-site advertising done for the screening protocol, VRC 000 (02-I-0127). Effort will be made to include women and minorities in proportions similar to that of the community from which they are recruited. Because this Phase I study is designed to establish safety of the vaccine in healthy adults, enrollment will be limited to persons at least 18 years of age and no older than 55 years of age.

8.7.1 Participation of Children

Children are not eligible to participate in this clinical trial because it does not meet the guidelines for inclusion of children in research. These guidelines (45 CFR 46, Subpart D, 401-409), state the Department of Health and Human Services protections for children who participate in research. Generally, healthy children can be studied when the research is considered as "not greater than minimal risk." Children can be involved in research with greater than minimal risk only when it presents the prospect of direct benefit to the individual child or is likely to yield generalizable knowledge about the child's disorder or condition.

8.8 Compensation

Subjects will be compensated for time and inconvenience in accordance with the standards for compensation of the Clinical Research Volunteer Program. The compensation per visit will be $275 for Day 0 visit that includes an injection and blood drawing, $175 for scheduled visits that include blood drawing (but $375 for Week 4 if an apheresis is performed) and $75 for visits for any unscheduled clinic visit which does not include a blood draw or procedure. The approximate total compensation for the subject will be about $975 based on the projected 5 clinic visits through Week 24, or $1,175 if an apheresis is done.

8.9 Safety Monitoring

Close cooperation between the designated members of the Protocol Team will occur to evaluate and respond to individual adverse events in a timely manner. The VRC designated Safety Officer for the day conducts a daily safety review of clinical data per VRC Standard Operating Procedures. Designated team members (Principal Investigator, Associate Investigators, Study Coordinator, Protocol Specialist, and other study clinicians) will review the summary study safety data reports on a weekly basis through 4 weeks after the last subject receives a study injection in order to be certain that the vaccine has an acceptable safety profile and will continue to monitor the study safety data reports on a monthly basis through completion of the last Week 24 visit. The DAIDS Medical Officer will provide an independent review of adverse events that have a bearing on study pausing (see Section 4.7).

9. ADMINISTRATIVE AND LEGAL OBLIGATIONS

9.1 Protocol Amendments and Study Termination

Protocol Amendments must be made only with the prior approval of the NIAID's Division of AIDS and Vaccine Research Center. Agreement from the investigator must be obtained for all protocol amendments and amendments to the informed consent document. All study amendments will be submitted to the IRB for approval.

The Division of AIDS, the Vaccine Research Center, the NIAID IRB, the Office of Human Research Protections, the Principal Investigator and the Food and Drug Administration reserve

the right to terminate the study. The investigator will notify the IRB in writing of the study's completion or early termination.

9.2 Study Documentation and Storage

The investigator will maintain a list of appropriately qualified persons to whom trial duties have been delegated.

Source documents are original documents, data, and records from which the subject's data are obtained. These include but are not limited to hospital records, clinical and office charts, laboratory and pharmacy records, microfiches, radiographs, and correspondence.

The investigator and staff are responsible for maintaining a comprehensive and centralized filing system of all study-related (essential) documentation, suitable for inspection at any time by representatives from the NIAID's Division of AIDS and Vaccine Research Center, IRB, FDA, and/or applicable regulatory authorities. Elements include:

- Subject files containing completed informed consent forms, and supporting copies of source documentation (if kept)
- Study files containing the protocol with all amendments, Investigator Brochures, copies of all correspondence with the IRB and the NIAID's Division of AIDS and Vaccine Research Center

In addition, all original source documentation must be maintained and be readily available.

All essential documentation should be retained by the institution for the same period of time required for medical records retention. The FDA requires study records to be retained for up to two years after marketing approval or refusal (21 CFR 312.62). No study document should be destroyed without prior written agreement between the NIAID's Division of AIDS and Vaccine Research Center and the investigator. Should the investigator wish to assign the study records to another party or move them to another location, they must notify the NIAID's Division of AIDS and Vaccine Research Center in writing of the new responsible person and/or the new location.

9.3 Study Monitoring, Data Collection and Data Monitoring

9.3.1 Study Monitoring

The NIAID's Division of AIDS and Vaccine Research Center regulatory authority inspectors or their authorized representatives are responsible for contacting and visiting the investigator for the purpose of inspecting the facilities and, upon request, inspecting the various records of the trial, provided that subject confidentiality is respected.

Site visits by study monitors will be made in accordance with the IND Sponsor (DAIDS) policy to monitor the following: study operations, the quality of data collected in the research records, the accuracy and timeliness of data entered in the database, and to determine that all process and regulatory requirements are met.

Site investigators will allow the study monitors, the NIAID IRB, and the FDA to inspect study documents (e.g., consent forms, drug distribution forms, case report forms) and pertinent hospital or clinic records for confirmation of the study data.

9.3.2 Data Collection

Clinical research data will be collected in a secure electronic data management system through a contract research organization, EMMES (Rockville, MD). Extracted data without patient identifiers will be sent to the Protocol Statistician for statistical analysis.

9.4 LANGUAGE

All written information and other material to be used by subjects and investigative staff must use vocabulary and language that are clearly understood.

9.5 POLICY REGARDING RESEARCH-RELATED INJURIES

The Clinical Center will provide short-term medical care for any injury resulting from participation in this research. In general, the National Institutes of Health, the Clinical Center, or the Federal Government will provide no long-term medical care or financial compensation for research-related injuries.

10. REFERENCES

1. UNAIDS. *AIDS Epidemic Update*. December 2007 [cited; Available from: http://data.unaids.org/pub/EPISlides/2007/2007_epiupdate_en.pdf].

2. *Approaches to the development of broadly protective HIV vaccines: challenges posed by the genetic, biological and antigenic variability of HIV-1: Report from a meeting of the WHO-UNAIDS Vaccine Advisory Committee Geneva, 21-23 February 2000.* AIDS, 2001. 15(6): p. W1-W25.

3. Nabel, G., W. Makgoba, and J. Esparza, *HIV-1 Diversity and Vaccine Development.* Science, 2002. 296(5577): p. 2335.

4. Osmanov, S., C. Pattou, N. Walker, B. Schwardlander, and J. Esparza, *Estimated global distribution and regional spread of HIV-1 genetic subtypes in the year 2000.* J Acquir Immune Defic Syndr, 2002. 29(2): p. 184-90.

5. Hemelaar, J., E. Gouws, P.D. Ghys, and S. Osmanov, *Global and regional distribution of HIV-1 genetic subtypes and recombinants in 2004.* AIDS, 2006. 20(16): p. W13-23.

6. Catanzaro, A.T., R.A. Koup, M. Roederer, R.T. Bailer, M.E. Enama, et al., *Phase 1 safety and immunogenicity evaluation of a multiclade HIV-1 candidate vaccine delivered by a replication-defective recombinant adenovirus vector.* J Infect Dis, 2006. 194(12): p. 1638-49.

7. Cohen, J., *Did Merck's Failed HIV Vaccine Cause Harm.* Science, 2007. 318(5852): p. 1048-9.

8. Jackson, L.A., G. Austin, R.T. Chen, R. Stout, F. DeStefano, et al., *Safety and immunogenicity of varying dosages of trivalent inactivated influenza vaccine administered by needle-free jet injectors.* Vaccine, 2001. 19(32): p. 4703-9.

9. Marshall, J.L., M.J. Hawkins, K.Y. Tsang, E. Richmond, J.E. Pedicano, et al., *Phase I study in cancer patients of a replication-defective avipox recombinant vaccine that expresses human carcinoembryonic antigen.* J Clin Oncol, 1999. 17(1): p. 332-7.

10. Marshall, J.L., R.J. Hoyer, M.A. Toomey, K. Faraguna, P. Chang, et al., *Phase I study in advanced cancer patients of a diversified prime-and-boost vaccination protocol using recombinant vaccinia virus and recombinant nonreplicating avipox virus to elicit anti-carcinoembryonic antigen immune responses.* J Clin Oncol, 2000. 18(23): p. 3964-73.

11. Meseda, C.A., R.R. Stout, and J.P. Weir, *Evaluation of a needle-free delivery platform for prime-boost immunization with DNA and modified vaccinia virus ankara vectors expressing herpes simplex virus 2 glycoprotein D.* Viral Immunol, 2006. 19(2): p. 250-9.

12. Merck & Co, I. *(WO/2001/002607) Adenovirus Carrying Gag Gene HIV Vaccine*. World Intellectual Property Organization 2001 [cited; Available from: http://www.wipo.int/pctdb/en/wo.jsp?WO=2001%2F02607&IA=WO2001%2F02607&DISPLAY=DESC.

13. Helms, T., B.O. Boehm, R.J. Asaad, R.P. Trezza, P.V. Lehmann, et al., *Direct visualization of cytokine-producing recall antigen-specific CD4 memory T cells in healthy individuals and HIV patients.* J Immunol, 2000. 164(7): p. 3723-32.

14. Betts, M.R., J.P. Casazza, and R.A. Koup, *Monitoring HIV-specific CD8+ T cell responses by intracellular cytokine production.* Immunol Lett, 2001. 79(1-2): p. 117-25.

15. Sprangers, M.C., W. Lakhai, W. Koudstaal, M. Verhoeven, B.F. Koel, et al., *Quantifying adenovirus-neutralizing antibodies by luciferase transgene detection: addressing preexisting immunity to vaccine and gene therapy vectors.* J Clin Microbiol, 2003. 41(11): p. 5046-52.

16. Butman, B.T., P.L. Gomez, R. Sheets, J. Gall, D. Brough, et al., *Manufacture and testing of a multi-clade adenoviral vector-based candidate vaccine against human immunodeficiency virus.* BioProcessing Journal, 2006. 5(2): p. 15-9.

17. Seth, P., *Adenoviruses : basic biology to gene therapy*. Medical intelligence unit. 1999, Austin, TX: R.G. Landes Co.

18. Brough, D.E., A. Lizonova, C. Hsu, V.A. Kulesa, and I. Kovesdi, *A gene transfer vector-cell line system for complete functional complementation of adenovirus early regions E1 and E4.* J Virol, 1996. 70(9): p. 6497-501.

19. Butman, B.T., A. Lizonova, D.E. Brough, J.M. Sowers, R. Sheets, et al., *Comprehensive characterization of the 293-ORF6 cell line.* Dev Biol (Basel), 2006. 123: p. 225-33; discussion 65-6.

APPENDIX I
STUDY INFORMED CONSENT FORM

INSTITUTE: Vaccine Research Center, National Institute of Allergy and Infectious Diseases

STUDY NUMBER: 08-I-0171 PRINCIPAL INVESTIGATOR: Barney S. Graham, M.D., Ph.D.

STUDY TITLE: VRC 015: A Phase I, Open-Label Clinical Trial to Evaluate the Safety, Tolerability and Immunogenicity of a Multiclade Recombinant HIV-1 Adenoviral Vector Vaccine, VRC-HIVADV014-00-VP, in Uninfected Adults Randomized to Needle or Biojector Methods of Intramuscular Injection

Latest IRB Review:
Latest Amendment Approved:
Study Consent: Version 5.0

INTRODUCTION

We invite you to take part in a research study at the National Institutes of Health (NIH).

First, we want you to know that:

> Taking part in NIH research is entirely voluntary.
>
> You may choose not to take part, or you may withdraw from the study at any time. In either case, you will not lose any benefits to which you are otherwise entitled. However, to receive care at the NIH, you must be taking part in a study or be under evaluation for study participation.
>
> You may receive no benefit from taking part. The research may give us knowledge that may help people in the future.

Second, some people have personal, religious or ethical beliefs that may limit the kinds of medical or research treatments they would want to receive (such as blood transfusions). If you have such beliefs, please discuss them with your NIH doctors or research team before you agree to the study.

Now we will describe this research study. Before you decide to take part, please take as much time as you need to ask any questions and discuss this study with anyone at NIH, or with family, friends or your personal physician or other health professional.

PURPOSE OF THE STUDY

The main purpose of this study is to evaluate the safety and tolerability of an experimental HIV vaccine when administered either by needle injection or using a needle-free device called the "Biojector". The needle-free device to be used in this study is the Biojector 2000®, which is "cleared" for use by the Food and Drug Administration (FDA) for delivery of vaccines into muscle. This study will see if these two methods result in similar or different side effects and immune system responses. The experimental study vaccine has been given to people before, but only in research studies in which it has been given by needle and syringe. This will be the first

time the study vaccine will be given by Biojector.

"Experimental" means that the study vaccine has not been approved by the FDA for treating or preventing HIV infection. The FDA allows it to be used in research studies only.

You are eligible to participate in this study because you have completed the screening process, completed an assessment of understanding, are not infected with HIV, are at low risk of becoming HIV infected based on the risk assessment performed during screening, do not have any significant medical problems, are an adult who meets the age requirement, and are willing to donate blood samples for future research.

About forty (40) people will participate in this study at the NIH Clinical Center in Bethesda, Maryland. Twenty (20) will be volunteers who have never received any HIV vaccine in a study before. As many as another twenty (20) will be volunteers who have participated in an HIV vaccine study before in which they received at least one injection of the study vaccine. The study visits will take about 24 weeks to complete. While on the study, you will be checked for vaccine side effects. You will be treated at the National Institutes of Health if any side effects occur. You will be asked to either return for an annual visit or to complete an annual contact by telephone or mail for 4 more years.

You will be informed of any new information learned during this study that might cause you to change your mind about staying in the study. At the end of the study, you will be told when study results may be available and how to learn about them.

Potential Risk of HIV Vaccine:

You should know that receiving an experimental HIV vaccine could increase (rather than decrease or not change) your risk of getting HIV, if you are later exposed to HIV (for example through sex or drug use). This may have been the case for certain participants in the Step Study. This study tested an HIV vaccine made by Merck, Inc. This vaccine included a common cold virus called adenovirus (type 5). This adenovirus was changed so it could not infect people. It was used to carry bits of HIV genes into the body.

The Step Study enrolled 1850 men and 1150 women at increased risk of HIV infection. Half got the Merck rAd5 vaccine and half got injections without the vaccine (placebo). Everyone got counseling about how to lower their risk of HIV infection. The study was stopped early for two reasons. The vaccine did not prevent HIV infection and it did not lower the amount of HIV virus in the blood. When the study was stopped, 82 men had gotten HIV infections; there were 49 HIV infections in the vaccine group and 33 in the placebo group. There was 1 women in the study who had HIV infection. Each case was reviewed carefully. The vaccine itself did **NOT** give anyone HIV.

In the Step Study, some men who got vaccine were more likely to get HIV. Two factors seemed to increase the risk of HIV infection in the vaccinated men compared to men who got placebo:

- having had an adenovirus (type 5) infection in the past before joining the study. (This is found by looking for "Ad5 antibody" in a blood test).

- having a foreskin on the penis (being uncircumcised).

Men with both of these factors had the highest risk of HIV infection. We do not yet know why.

One (1) woman had HIV in the Step Study at the time it was stopped. A study of the Merck rAd5 vaccine in South Africa (called "Phambili") was stopped early. There were some HIV infections in women who got vaccine and in women who got placebo. The numbers are small, so Phambili cannot be analyzed in the same way as the Step Study. It is possible that vaccinated women could be at increased risk of HIV infection.

The VRC rAd5 vaccine is similar in some ways and different in other ways from the Merck rAd5 vaccine.

In the VRC 015 study, only people with a low risk of HIV infection will be enrolled. **It is important for you to continue to avoid behaviors associated with the risk of getting HIV infection**.

STUDY VACCINE

Vaccines are substances used to try to create an immune response (the body's natural defenses) to prevent or resist an infection. There is no live HIV virus in the study vaccine. The vaccine itself cannot cause you to become infected with HIV. You must be exposed to the HIV virus (for example through sex with an HIV-infected person) to become infected with HIV.

The experimental HIV vaccine in this study is known as VRC-HIVADV014-00-VP. In this consent document, we call it the "rAd5 vaccine" or "study vaccine."

Adenovirus is a common virus that causes upper respiratory infections (such as the common cold), eye infection (conjunctivitis), urine infection or diarrhea. The rAd5 vaccine is a modified adenovirus that will carry DNA into cells in the body. The rAd5 vaccine has codes for parts of the three HIV proteins called Gag, Pol, and Env. The manufactured DNA has been packaged in an adenovirus shell that is missing some of the usual adenovirus genes. It cannot reproduce in a human body. You cannot infect someone else with the study vaccine adenovirus.

The study vaccines are manufactured by packaging DNA into an adenovirus shell, similarly to the Merck vaccine. The study vaccines code for a different HIV protein than was in the Merck vaccine. An adenovirus with DNA inserted in a laboratory is called a vector or adenoviral vector.

STUDY PROCEDURES

The study will have two groups. Group 1 will have twenty people in it that have never received the study vaccine before. Group 2 will have up to twenty people who received one or more injections of the study vaccine before in another protocol. In each group, half of the people will get their injection by the Biojector and half by the more commonly used needle and syringe.

VRC 015	Subgroup	Number of participants	Day 0 Injection
Group 1 (never received the study vaccine before)	1a	10	needle
	1b	10	Biojector
Group 2 (received at least one injection of the study vaccine before)	2a	5 to 10	needle
	2b	5 to 10	Biojector
Total number to be enrolled		30 to 40	

Before the study started each study number was randomly assigned (like flipping a coin) to either the Biojector or the needle and syringe as the type of injection. You will have an equal chance of being assigned to get your injection by Biojector or needle and syringe.

The injection will be given into muscle in your upper arm. You will get your vaccination on the day you enroll in the study. This is called Day 0. The clinic staff will observe you for at least 30 minutes after the study injection. You will be asked to keep track of your symptoms at home for 5 days after the study injection.

After the injection, you must come to the clinic if you have a rash, hives, fever of 101.6˚F or higher, or a lot of difficulty in daily activities (such as going to work or taking care of yourself). You will also need to come to the clinic for any problem that the nurse or doctor thinks should be checked by exam or blood tests or urine tests. It is very important that you follow the instructions given to you by the study nurse.

If your injection site develops a bump, bruise, scab or other skin change, then a photo may be taken to keep a record of the problem. Your identity will not be shown in the photo. You may refuse to have a photo. Study investigators will use the photographs to learn more about vaccine reactions.

You will have about 5 clinic visits and a phone call follow-up over 24 weeks if you enroll in this study. The visit schedule is shown in the table below. The vaccination visit will take about 4 hours to complete. Clinic visits with a blood collection will usually take about 2 hours, but may be shorter. The Week 4 visit may take about 3 hours if you have an apheresis procedure. The apheresis procedure is explained in detail in the next section of this consent.

	Day 0	Day 2	Week 2	Week 4	Week 12	Week 24	Annual Contact for 4 yrs.
Assessment of Understanding & Informed Consent	X						
Vaccine Injection	X						
Telephone contact (or Clinic Visit)		X					X
5-day Diary Card	Begin		Review with Nurse				
Clinical Evaluation (vital signs, weight); physical exam if needed	X		X	X	X	X	
Medical history	X		X	X	X	X	
Blood Sample collection	X		X	Blood Draw or *Apheresis	X	X	**X
Pregnancy Test (for females)	X					X	
Counseling as needed about HIV and pregnancy avoidance	X		X	X	X	X	
HIV Tests	X					X	**X
Social Impact Questionnaire						X	
*Apheresis at Week 4 is an option available only to Group 2 participants.<br>**Done if the long-term follow-up is by clinic visit							

At each visit, you will be checked for any health changes or problems. You will be asked how you are feeling and if you have taken any medications or supplements, including those that are not prescribed by a doctor. If it is not an emergency, call a study nurse or doctor before starting new medicines or getting other vaccines or shots of any type.

Blood will be drawn at some study visits to check on your health. You will be told right away if any of your test results show a health problem. Some blood samples will be used to study your immune response to the vaccine.

The amount of blood drawn will vary from about 1 tablespoon (15 mL) to about 10 tablespoons (150 mL), depending on the visit. The total amount of blood drawn during the 24 weeks of study clinic visits is estimated to be about 2 ½ cups (550 mL).

You will be tested for HIV several times and asked questions about your sexual behavior and drug use. Throughout the study you will be counseled on how to lower your risk of getting HIV. You will be asked about any social effects you may have experienced from your participation in this study.

The vaccine used in this study cannot cause HIV infection. However, if you become infected with HIV during this study for other reasons, you will be referred for medical care. Medical treatment for HIV infection is not part of this study. You will be asked to continue with study follow-up visits through the 24 weeks of the study.

You will be contacted at least once per year for four years after the last study clinic visit. You will have a choice of completing the annual contact as a clinic visit or contact by telephone, e-mail, or mail to answer questions about your health. If you come to the clinic you will be asked to give blood samples for research and HIV testing. About seven tablespoons of blood (107 mL) will be collected.

Apheresis

If you are in Group 2 (received the study vaccine before), then at the Week 4 visit, we would like to collect your blood sample by apheresis. In this procedure, blood will be removed through a needle in the vein of one arm, spun in a machine that permits separation of the desired blood component (white blood cells or plasma), and then the remainder will be returned through the same needle. Citrate, a medication to prevent blood from clotting, will be added to the blood while in the machine to prevent it from clotting. This procedure will be done at the Department of Transfusion Medicine in the NIH Clinical Center.

The purpose of the apheresis in this study is to allow the investigator to obtain a larger number of white blood cells than can be collected by simple blood drawing. Getting a lot of white blood cells will allow more laboratory tests to be done to see how the immune system responds to the study vaccine. The number of white blood cells collected is a small fraction of the total amount in your body. The body quickly replaces removed cells. Similar procedures are used on a daily basis in Blood Banks as a way of getting blood products from normal donors and as a type of therapy for certain diseases. The procedure will take approximately 1-3 hours.

Before apheresis is done, your weight, pulse and blood pressure will be checked. The result of your blood test at Week 2 is one factor in whether you are eligible for apheresis at Week 4. You will be asked questions about your general health and medical history. You will be asked to sign the Department of Transfusion Medicine's consent form for the apheresis procedure on the day of apheresis. You will be asked to lie on a recliner or couch. The kits used to collect the apheresis products are sterilized, single-use, disposable sets that are not in contact with any person's body fluids other than yours. No blood products are given to you during these procedures. A physician from the Department of Transfusion Medicine will be available in or near the apheresis donor area at all times.

If you do not wish to have apheresis or if you are not eligible for apheresis or if we cannot get an apheresis appointment for your Week 4 visit, the blood sample will be collected as usual in blood collection tubes using a needle

MONITORING OF THE STUDY

This study will be monitored by a group of physicians and scientists at the Clinical Center. This group will review the information from the study and will pay close attention to any serious side effects. If serious side effects occur, further injections may be delayed or canceled.

HIV TESTING

We will test your blood for HIV, the virus that causes AIDS. If you have HIV, we will explain what it means for you. If you live in Maryland, the NIH Clinical Center will report your name and HIV results to the Maryland Department of Health and Mental Hygiene.

We will tell you the results of your HIV tests throughout the study. If you have any questions regarding the HIV testing, you are encouraged to discuss them with the study nurse or doctor, or you may call a Clinical Center HIV counselor at 301-496-2381.

GENETIC TESTING

In the future, genetic research tests to help understand how vaccines work may be done on your DNA using stored samples. In vaccine research some genetic tests are done to see if different types of immune response to a vaccine seem to be related to genetic differences in people. Genetic tests done in a research lab from your stored samples will not be in your medical record and will not have your name on the sample.

Some genetic tests are done in a regular medical laboratory. HLA type is a genetic test ordered through the NIH Clinical Center medical laboratory. HLA type results will be in your medical record at the NIH Clinical Center.

People with certain HLA types might be more likely to develop certain diseases. Simply having those HLA types doesn't mean they will develop those diseases. It is our policy not to discuss your HLA results unless they have direct medical or reproductive implications for you or your family. The results of these tests are not used to make health care decisions.

STORED SAMPLES

Some of the blood samples collected from you will be stored for future research to learn more about HIV and HIV vaccines, the immune system, and/or other medical conditions.

The results from the research done with your stored samples will not be given to your health care provider and will not be put in your medical record.

Labeling of Stored Samples

Your stored samples will be labeled by a code (such as a number) that only the study team can link to you. Any identifying information about you will be kept confidential to the extent permitted by law.

Risks from Stored Samples

The greatest risk is the unplanned release of information from your medical records. The chance that this information will be given to an unauthorized person without your permission is very

small. Possible problems with the unplanned release of information include discrimination when applying for insurance and employment. Similar problems may occur if you disclose information yourself or agree to have your medical records released.

Future studies

In the future, other investigators (at NIH or outside of NIH) may wish to study your stored samples. When the study team shares your materials, they may share it with no identifying information or with a code. Some information about you, such as your gender, age, health history, or ethnicity may also be shared with other investigators. Any future research studies using your samples will be reviewed by the investigator's Institutional Review Board (IRB), a special committee that oversees medical research studies to protect the rights and welfare of human subject volunteers.

Your stored materials will be used only for research and will not be sold. The research done with your materials may be used to develop new products in the future but you will not receive payment for such products.

Making your Choice

Refusal to let us store your samples means you are not eligible to be in this specific study. If you agree to participate in this study, this means you also agree to let us store your samples for future research. Your decision does not affect your eligibility for other studies at NIH. Even if you agree now to let us store your samples, you can change your mind later. If you do, please contact us.

POSSIBLE STUDY RISKS

Injection Risks

It is possible that you may have some side effects from the injection. You will receive your rAd5 vaccine injection either with a needle and syringe or with a needle-free device called a "Biojector." Injection risks are similar with both devices, and include stinging, arm discomfort, pain, soreness, redness, bruising, swelling or a small laceration (cut).

General Vaccine Risks

It is possible to have one or more of the following side effects: fever, chills, rash, aches and pains, nausea, headache, dizziness, or fatigue. As with all vaccines or drugs, you could have an immediate allergic reaction, including a rash, hives, or even difficulty breathing. Allergic reactions can be life threatening; therefore, the clinic staff will watch you for at least 30 minutes after each immunization and provide any needed treatment. There may be other side effects, even serious ones that we don't know about yet. Therefore, it is important that you report any side effects to the clinic staff as soon as they occur.

Adenoviral Vector (rAd5) Vaccine Risks

VRC Adenoviral Vector Vaccine: The first study of the VRC rAd5 vaccine started at the VRC

Clinic in July 2004. Other larger studies are ongoing in international sites. More than 140 people have received this vaccine in studies at the NIH Clinical Center and more than 800 people in studies done elsewhere.

The VRC rAd5 vaccine has been safe in studies so far. After a rAd5 vaccination, some people have a flu-like condition with fever, headache, muscle aches, tired feeling and chills. It starts about 12-16 hours after vaccination and lasts a few hours. Some people have injection site pain or discomfort in the first few days after a vaccination. The flu-like symptoms and injection site pain or discomfort may be treated with an over-the-counter medicine for pain and fever. About 5% of people have experienced redness and swelling at the injection site (up to 4-5 inches in diameter) beginning around 5 to 7 days after the injection. The appearance of a large red area around the injection site resolved within a few days.

Sometimes a small red bump and then a scab have been seen to form at the injection site of a rAd5 vaccination. The scab was less than ½ inch across. It was not deep and it was not infected. The scab came off after a few days. The skin healed without needing any treatment. In current studies this was seen when the vaccine was administered just under the skin.

During the study, regular blood tests and check-ups will be performed to check for possible side effects. Some blood will be stored during the study in case additional safety tests are needed.

<u>Adenovirus antibodies</u>: You may develop antibodies to adenovirus type 5 from the rAd5 vaccine. It is possible you would not be able to receive (or have a reduced response to) future investigational products that use an adenoviral vector. Currently, there are no products approved by the FDA that use an adenoviral vector. The Step Study results did not suggest that Ad5 antibodies from vaccine increase your risk of HIV infection if you are ever exposed to HIV in the future.

Risks from Blood Drawing

Blood will be drawn from a vein in your arm using a needle. Blood drawing may cause pain and bruising and, rarely, infection at the place where the blood is taken. Sometimes drawing blood causes people to feel lightheaded or even faint.

Other Risks of Being in an HIV Vaccine Study

Getting the experimental HIV vaccine in this study may mean that you cannot be in other experimental HIV vaccine studies later. It is also possible that receiving the experimental HIV vaccine may alter your response to future HIV vaccines and may make them either more or less effective. If you are exposed to HIV through sex or drug use after receiving the study injection, your risk of becoming HIV infected is unknown. Please do not do anything that might expose you to HIV.

You should be aware that some people who received experimental HIV vaccines in the past became infected with HIV through sex or drug use. We know that HIV infection and AIDS can develop even in a person who has received a test vaccine if he/she is exposed to HIV. If you are exposed to HIV through sex or drug use after receiving the study vaccine, your risk of becoming

infected with HIV and developing AIDS is unknown. Please inform the VRC Clinic any time you think that you may have been exposed to HIV. If you do get infected, we do not know what effect the study vaccine may have on the disease. The time that it takes for you to become sick from HIV/AIDS may be the same, longer, or shorter than usual. You will be educated and counseled about HIV exposure often during the study. If you have questions, please ask the clinic staff.

Risk of Developing a "False" Positive HIV Test

At the time you enroll in the study you must have a negative HIV antibody test. An HIV antibody test (called an ELISA or Western Blot) is the usual way to test for HIV infection. <u>After the study vaccinations, it is very likely that you will test positive for HIV antibody from the study vaccine.</u> However, it will be possible, by using tests for the presence of HIV virus (called PCR or viral load testing), to show when a positive result on the HIV antibody test is NOT because of an HIV infection. A positive antibody test in a person who is not HIV infected is called a "false positive" test. If you do have a false positive HIV antibody test caused by the study vaccine, it is unknown how long the test will be positive. You cannot pass antibodies to your partner.

If you have a false positive antibody test at the end of the study you are encouraged to be retested at the VRC Clinic before taking the test for insurance, travel or other purposes. You may have HIV testing at the VRC Clinic either through visits for this study or through a VRC sample collection study (depending upon whether the testing is needed during or long after study completion) so that you can find out if it changes back to negative. There are social risks from having your HIV test appear positive. For this reason you are advised to have all HIV testing done at the VRC Clinic. Counseling about HIV tests, including social problems related to false positive results, is offered at all clinic visits. You may also call the clinic at other times if you have questions or concerns.

Any time you have a positive HIV antibody test in the future, you must also have an HIV "viral load" (PCR) test. Otherwise, you and others will not know if the positive HIV antibody test is from the study vaccine or from HIV infection.

You will not be able to donate blood while you are participating in the study and for at least one year after the last study injection. You may not be able to donate blood ever again if you have a false positive HIV antibody test when you try to donate blood. Please be sure to ask the VRC Clinic to see if you have a negative HIV antibody test before trying to donate blood.

If you have a false positive antibody response on HIV tests, you may also have difficulties with:

- Health insurance
- Life Insurance
- Medical or dental care
- Travel to other countries or immigration
- Employment

- Education
- Housing
- Military services or other government agencies
- Personal relationships

If you have problems like these, the staff at the clinic will try to help you work through them. If your blood tests look HIV positive because of study vaccinations you will be offered a letter that shows you joined this study and that describes the antibody response caused by the vaccine. Even so, this letter or other help offered by the VRC Clinic may not solve a social problem caused by a false positive HIV antibody test.

It is also possible that others may learn that you are taking part in this study and assume that you are at risk of HIV infection because of sexual behavior or drug use. This may result in some people treating you differently.

Risks from Pregnancy

We do not know the possible effects of the study vaccine on the fetus or nursing infant. Women must also agree to practice adequate birth control beginning at least 21 days prior to receiving the injection until the last study visit at Week 24, or not be able to have children. Birth control includes: condoms, male or female, with or without a spermicide; diaphragm or cervical cap with spermicide; intrauterine device; birth control pills or patch; Depo-Provera; and other prescription methods; or a male partner who has previously undergone a vasectomy. Women must have a negative pregnancy before each study injection. You must notify the clinic staff right away if you have become pregnant during this study or think that you might be pregnant. If you become pregnant, you will be asked to continue with study follow-up visits through the full 24 weeks of study visits, as well as to report the outcome of the pregnancy.

Apheresis Procedure Risks

Apheresis donations are generally safe and side effects are rare. Pain, bruising or discomfort at the needle placement site may occur. Sometimes apheresis causes a tingling sensation around the mouth or in the finger, chills, nausea, heartburn or mild muscle cramps. This can usually be relieved by slowing or temporarily interrupting the apheresis or taking a calcium containing antacid, such as Tums®. Other possible side effects are anxiety, vomiting and lightheadedness. Temporary lowering of the blood pressure may develop. There is the rare possibility of infection, fainting or seizure. Very rarely a nerve problem at the needle placement site may occur. Also, very rarely, a machine malfunction may occur, resulting in the loss of about one unit (one pint) of blood.

There are theoretical risks from re-infusion of the blood after processing by the machine such as infection or an adverse reaction to the blood components. However, this has not been seen in many thousands of volunteers who have undergone this or similar procedures to date. Rarely the performance of frequent apheresis procedures over a short period of time can result in a drop in total blood cell counts. This risk should not apply to this study because it includes only one possible apheresis.

There may be additional risks of apheresis that are unknown at this time. Any new information that may affect your willingness to participate in this study will be disclosed to you.

POSSIBLE BENEFITS

This study may be of no direct benefit to you because no one knows if vaccines against HIV work. However, you and others may benefit in the future from the information that will be learned from the study.

COSTS TO YOU FOR YOUR PARTICIPATION

You do not have to pay for the vaccine, research clinic visits, examinations or laboratory tests that are part of this study. All medical costs outside this study will be paid by you or your health insurance carrier (if you have insurance).

PAYMENT TO YOU FOR YOUR PARTICIPATION

You will be compensated $275 for the visit that includes an injection and blood drawing. You will be compensated $175 for each visit that includes blood drawing, but the compensation for the visit will be $375 if the apheresis procedure is performed. You will be compensated $75 for any unscheduled clinic visit which does not require blood drawing or a procedure. The approximate total compensation for the 24 weeks of study will be about $975 for Group 1. For Group 2 it will be about $975 if apheresis is not done or $1,175 if apheresis is done at Week 4. Actual compensation is based on the number and type of study visits you complete. You will be paid throughout the study by checks, which will be mailed to you after each reimbursable visit.

REASONS FOR REMOVING YOU FROM THE STUDY WITHOUT YOUR CONSENT

You may be asked to leave the study for several different reasons, including:

- You don't keep appointments or follow study procedures
- The study sponsor or study doctor decides to stop or cancel the study
- The Institutional Review Board or the FDA decide that the study should be stopped

If you agree to take part in this study, it is important for you to keep all your appointments. If you want to stop participating, you may stop at any time. However, continuing to attend the study visits for safety check-ups is for your benefit. It is important to check your health for the time period stated in the study protocol.

ALTERNATIVES

You may choose to not participate in any HIV vaccine study. You may be eligible for other studies, including those testing other experimental HIV vaccines. Your study doctor can discuss the risk and benefits of alternative studies.

COMMUNITY RESOURCES

You may also be interested in contacting local volunteer panels of individuals from the general public that were organized to assist and advise AIDS vaccine trials in the metropolitan Washington DC area, the Capital Area Vaccine Effort (CAVE) and the Community Advisory Board (CAB). Information is available at the Internet site http://www.aidsvaccine.org.

CONFLICT OF INTEREST

The National Institutes of Health reviews NIH staff researchers at least yearly for conflicts of interest. The following link contains details on this process http://ethics.od.nih.gov/forms/Protocol-Review-Guide.pdf .You may ask the research team for additional information or a copy of the Protocol Review Guide.

The National Institutes of Health, including some members of the Vaccine Research Center scientific staff, developed the investigational vaccine being used in this research study. The results of this study could play a role in whether the FDA will approve the vaccine for sale at some time in the future. If approved, the future sale of the vaccine could lead to payments to NIH and some NIH scientists. By U.S. law, government scientists are required to receive such payments for their inventions. You will not receive any money from the development or sale of the product.

One or more investigators participating in this study may have less than $15,000 of stock in the manufacturer of the product used in this study. Under federal regulations, however, this is permissible and does not create a conflict of interest.

This protocol may have investigators who are not NIH employees. Non-NIH investigators are expected to adhere to the principles of the Protocol Review Guide but are not required to report their personal financial holdings to the NIH.

OTHER PERTINENT INFORMATION

1. Confidentiality. When results of an NIH research study are reported in medical journals or at scientific meetings, the people who take part are not named and identified. In most cases, the NIH will not release any information about your research involvement without your written permission. However, if you sign a release of information form, for example, for an insurance company, the NIH will give the insurance company information from your medical record. This information might affect (either favorably or unfavorably) the willingness of the insurance company to sell you insurance.

The Federal Privacy Act protects the confidentiality of your NIH medical records. However, you should know that the Act allows release of some information from your medical record without your permission, for example, if it is required by the Food and Drug Administration (FDA), members of Congress, law enforcement officials, or other authorized people.

2. Policy Regarding Research-Related Injuries. The Clinical Center will provide short-term medical care for any injury resulting from your participation in research here. In general, no long-term medical care or financial compensation for research-related injuries will be provided by the National Institutes of Health, the Clinical Center, or the Federal Government. However, you have the right to pursue legal remedy if you believe that your injury justifies such action.

3. Payments. The amount of payment to research volunteers is guided by the National Institutes of Health policies.

4. Problems or Questions. If you have any problems or questions about this study or about any research-related injury, contact the Principal Investigator, Barney S. Graham, M.D., Ph.D. at 301-594-8468, or the Study Coordinator, Laura Novik, RN at 301-451-8717 or 1-800-NIH-BEEP ext 14881.

If you have any questions about your rights as a research subject, you may call the Clinical Center Patient Representative at 301-496-2626.

5. Consent Document. Please keep a copy of this document in case you want to read it again.

COMPLETE APPROPRIATE ITEM(S) BELOW:

Adult Study Participant's Consent

I have read the explanation about this study and have been given the opportunity to discuss it and to ask questions. I hereby consent to take part in this study.

_______ Time

____________________________________ ________
Signature of Adult Participant/Legal Representative Date

THIS CONSENT DOCUMENT HAS BEEN APPROVED FOR USE FROM XXXXXX THROUGH XXXXXX.

Time: ________		Time: ________	
Signature of Investigator/Person Obtaining Consent	Date	Signature of Witness	Date

APPENDIX II
CONTACT INFORMATION

Principal Investigator:
Barney S. Graham, M.D., Ph.D. 301-594-8468
Vaccine Research Center, NIAID, NIH
40 Convent Drive, MSC 3017
Bethesda, MD 20892-3017

Associate Investigators:
Joseph Casazza, M.D., Ph.D. 301-594-8627
Cynthia Starr Hendel, CRNP 301-451-8715
LaSonji Holman, FNP 301-402-8641
Sarah Plummer, RN, MSN, NP 301-402-8640
Julie Ledgerwood, D.O. 301-594-8502
Uzma Sarwar, M.D. 301-402-9043
LeeJah Chang, M.D., 301-451-9644

Study Coordinators/Research Nurses
Laura Novik, RN, MA, CCRC
VRC 015 Study Coordinator
All Clinic Staff: 301-451-8715
Pamela Costner, RN
Ingelise Gordon, RN
Brenda Larkin, RN, BSN, CCRC
Floreliz Medoza, RN
Jamie Saunders, RN,BSN
Kathy Zephir, RN, BSN, MS

DAIDS Medical Officer:
Elizabeth Adams, M.D., 301-435-3730
6700 B Rockledge Dr., Bethesda, MD 20892

Protocol Statistician:
Martha Nason, Ph.D., 301-451-5134
Biostatistical Research Branch, NIAID, NIH

NIH Apheresis Clinic
Susan Leitman, M.D. 301-496-9703
Department of Transfusion Medicine
10 Center Drive, Building 10-MSC 1184
Bethesda, MD 20892-1184

Data Coordinating Center:
Vaccine Research Center, NIAID, NIH and
EMMES Corporation, Rockville, MD

Study Site:
National Institutes of Health Clinical Center
Vaccine Evaluation Unit, CRC, 5-NES
Bethesda, MD 20892

Site And Data Monitoring:
PPD, Wilmington, NC

Scientific and Laboratory Collaborators:
Gary Nabel, M.D., Ph.D.
Vaccine Research Center, NIAID, NIH
40 Convent Drive, MSC 3017
Bethesda, MD 20892

Laboratory of Immunology
VRC/NIAID/NIH:
Robert Bailer, Ph.D., 301-594-8481
Richard Koup, M.D., 301-594-8585
John Mascola, M.D., 301-594-8490
Mario Roederer, Ph.D., 301-594-8491

Research Immunology Central Laboratory:
NVITAL (NIAID Vaccine Immune T-Cell and Antibody Laboratory)
9 West Watkins Mill Road, Suite 150
Gaithersburg, MD 20878

Vaccine Manufacturer:
GenVec, Incorporated
65 Watkins Mill Road
Gaithersburg, MD 20878

Pharmacy:
Judith Starling, R.Ph., 301-496-4363
Hope DeCederfelt, R.Ph.
Pharmaceutical Development Section
Clinical Center, Building 10/1N257
Bethesda, MD 20892

VRC Production and Regulatory Affairs:
Richard Schwartz, Ph.D., 301-594-8485
Judy Stein, MPH, MBA, 734-763-7753
Michelle Conan-Cibotti, Ph.D., 301-451-2740

VRC Protocol Operations:
Mary E. Enama, M.A., PA-C, 301-594-8501
Galina Yamshchikov, M.S., 301-594-1064
Iris Pittman, BA, CCRP, 301-451-8543

DAIDS, Regulatory Support Center:
SAE Phone: 1-800-537-9979 or 301-897-1709
SAE Fax: 1-800-275-7619 or 301-897-1710
SAE e-mail: DAIDSRSCSafetyOffice@tech-res.com
Protocol Registration
Fax: 1-800-418-3544 or 301-897-1701
Phone: 301-897-1707
e-mail: protocol@tech-res.com

APPENDIX III
SCHEDULE OF EVALUATIONS

	**VRC 000 Screen**		**VRC 015: rAd5 Vaccination and Follow-up Visits**						Long-term Contacts
Visit		*01	02	02B	02C	03	04	05	06, 07, 08, 09
Week of Study			Wk 0	W 1	W 2	W 4	W 12	W 24	W 76, 128, 180, 232
[1]Day of Study			D 0	D 2	D 7	D 28	D 84	D 168	
**Clinical**									
VRC000 Screening consent		X							
VRC 015 AoU & Consent			X						
[2] Physical exam		X	[X]		[X]	[X]	[X]	[X]	
Complete or interim med hx; vital signs, weight through visit 05; specified long-term follow-up visits 06 to 09		X	X		X	X	X	X	X
Study Injection			X						
5-day Diary Card			start		review				
[3]Phone evaluation (clinic visit if needed)				X					
[4] Social Impact Assessment (SIA)								X	
[4] Counsel HIV; pregnancy		X	X		X	X	X	X	
CBC, differential, platelets	Lav.	3	3		3	3	3	3	
PT, PTT	Blue	5							
[5] Preg test: urine (or serum)		X	X					X	
Creatinine and ALT	SST	4	4		4	4	4	4	
HBsAg, Anti-HCV, HCV PCR	SST	8							
HLA class I, II antigens	ACD				20				
ELISA/Western Blot	SST	8	8					8	[8]
HIV PCR	Lav	3	3					3	[3]
RPR	SST	4							
**Research**									
Adenovirus Serology	SST	4	X			X		X	
HIV-specific antibody	SST		16			16		16	
ICS and ELISpot; PBMC & Plasma for Storage	EDTA	60	80			[6]Apheresis & 10 mL or 80 mL		80	[80]
Serum Storage	SST	24	16			16		16	[16]
**Daily Volume (mL)**		**123**	**130**		**27**	**119**	**7**	**130**	[6] **[107]**
**Cumulative Volume (mL)**		**123**	**253**		**280**	**399**	**406**	**536**	

* VRC 000 Screening evaluations may be completed over several screening visits. Each evaluation must be in the specified window for the test. Pregnancy test on Day 0 must be used for eligibility. If clinical assessment on Day 0 suggests significant changes since the screening visit, then physical exam, hematology, ALT and creatinine done on Day 0 must be used for eligibility.

[1] Day 0=day of enrollment and first injection; Day 0 evaluations prior to first injection are the baseline for assessing adverse events subsequently. The "A" visits (not shown) are the evaluations (vital signs and injection site assessment) completed between 30 to 45 minutes after a study injection. The "B" phone evaluations are 2(±1) days after rAd5; the "C" visit is 7 (±1) days after rAd5. Visit 03 is Week 4 (-3 to +7 days), visit 04 is Week 12±7 days and visit 05 is Week 24±7 days. Attempt to complete the long-term follow-up contacts (Visits 06, 07, 08, 09) within a ±28 day window

[2] Screening visit includes a physical exam; during other visits a physical exam is done if indicated by interim history or laboratory test results (shown as [X] in the table).

[3] Clinic visit is required if there is evidence of rash, urticaria (hives), fever of 38.7˚C (Grade 2) or higher that does not resolve within 24 hours, skin lesion formation, or significant impairment in the activities of daily living (ADL).

[4] SIA required at Week 24, but may be completed at any time subject indicates experiencing a social impact. The HIV and pregnancy prevention counseling is performed at Screening and on Day 0 and offered on each subsequent visit.

[5] Negative pregnancy test results must be confirmed for women of reproductive potential prior to administering study injections.

[6] At 03, PBMC will be collected by apheresis from Group 2 subjects whenever possible along with plasma from one 10 mL blood sample; collect 80 mL blood by phlebotomy to obtain the PBMC and plasma from Group 1 subjects and Group 2 subjects who are not having apheresis. Long-term follow-up blood draws (visits 06, 07, 08, 09) are optional; this is shown by brackets [ ]. HIV testing may continue to be done as needed for health assessments, but beginning with protocol Version 5.0 the stored research samples should not be collected unless requested by the Principal Investigator.

APPENDIX IV
TABLE FOR GRADING SEVERITY OF ADVERSE EVENTS

The Division of AIDS Table for Grading the Severity of Adult and Pediatric Adverse Events, Version 1.0, Dec 2004 (with Clarification, August 2009)

The table for Grading Severity of Adverse Events in this protocol is found on the Division of AIDS Regulatory Support Center (RSC) website:

http://rsc.tech-res.com/safetyandpharmacovigilance/

A complete copy of this table will be provided to the IRB for reference with initial review of the protocol. The table cannot be changed except by the IND Sponsor, DAIDS.

The full text of the table will also be included in the Protocol Manual for reference by the study clinicians who are assessing adverse events.
